# The Gutenberg health study: a five-year prospective analysis of psychosocial working conditions using COPSOQ (Copenhagen psychosocial Questoinnaire) and ERI (effort-reward imbalance)

**DOI:** 10.1186/s12889-021-12240-3

**Published:** 2022-01-06

**Authors:** Matthias Nuebling, Janice Hegewald, Karla Romero Starke, Hans-Joachim Lincke, Sylvia Jankowiak, Falk Liebers, Ute Latza, Stephan Letzel, Merle Riechmann-Wolf, Emilio Gianicolo, Manfred Beutel, Norbert Pfeiffer, Klaus Lackner, Thomas Münzel, Philipp S. Wild, Andreas Seidler

**Affiliations:** 1 FFAW: Freiburg Research Centre for Occupational Sciences, Bertoldstr. 63, D-79098, Freiburg, Germany; 2 Institute and Policlinic of Occupational and Social Medicine (IPAS), Faculty of Medicine Carl Gustav Carus, TU Dresden, Dresden, Germany; 3 Division Work and Health, Federal Institute for Occupational Safety and Health BAuA, Berlin, Germany; 4 Institute of Occupational, Social, and Environmental Medicine, University Medical Center of the Johannes Gutenberg University of Mainz, Mainz, Germany; 5 Institute for Teachers’ Health, University Medical Center of the Johannes Gutenberg University of Mainz, Mainz, Germany; 6 Institute of Medical Biostatistics, Epidemiology, and Informatics, University Medical Center of the Johannes Gutenberg University of Mainz, Mainz, Germany; 7 Institute of Clinical Physiology, National Research Council, Lecce, Italy; 8 Department of Psychosomatic Medicine and Psychotherapy, University Medical Center Mainz, Johannes Gutenberg University Mainz, Mainz, Germany; 9 Department of Ophthalmology, University Medical Center Mainz, Johannes Gutenberg University Mainz, Mainz, Germany; 10 Institute for Clinical Chemistry and Laboratory Medicine, University Medical Center Mainz, Mainz, Germany; 11 Center of Thrombosis and Hemostatis (CTH), University Medical Center Mainz, Mainz, Germany; 12 Center for Cardiology – Cardiology I, University Medical Center of the Johannes Gutenberg University of Mainz, Mainz, Germany; 13 Preventive Cardiology and Preventive Medicine, Center for Cardiology, University Medical Center of the Johannes Gutenberg University of Mainz, Mainz, Germany; 14 DZHK (German Center for Cardiovascular Research), Partner Site Rhine-Main, Mainz, Germany

**Keywords:** Psychosocial factors, Risk assessment, Longitudinal study, Prospective study, Prediction, Satisfaction, Health, COPSOQ, ERI

## Abstract

**Background:**

Psychosocial working conditions were previously analyzed using the first recruitment wave of the Gutenberg Health Study (GHS) cohort (*n* = 5000). We aimed to confirm the initial analysis using the entire GHS population at baseline (*N* = 15,010) and at the five-year follow-up. We also aimed to determine the effects of psychosocial working conditions at baseline on self-rated outcomes measured at follow-up.

**Methods:**

At baseline, working GHS participants were assessed with either the Effort-Reward-Imbalance questionnaire (ERI) (*n* = 4358) or with the Copenhagen Psychosocial Questionnaire (COPSOQ) (*n* = 4322); participants still working after five years received the same questionnaire again (ERI *n* = 3142; COPSOQ *n* = 3091). We analyzed the association between working conditions and the outcomes job satisfaction, general health, burnout, and satisfaction with life at baseline, at follow-up and also prospectively from baseline to follow-up using linear regression models. We examined the outcome variance explained by the models (R^2^) to estimate the predictive performance of the questionnaires.

**Results:**

The models’ R^2^ was comparable to the original baseline analyses at both t0 and t1 (R^2^ range: ERI 0.10–0.43; COPSOQ 0.10–0.56). However, selected scales of the regression models sometimes changed between assessment times. The prospective analysis showed weaker associations between baseline working conditions and outcomes after five years (R^2^ range: ERI 0.07–0.19; COPSOQ 0.07–0.24). This was particularly true for job satisfaction. After adjusting for the baseline levels of the outcomes, fewer scales still explained some of the variance in the distribution of the outcome variables at follow-up. The models using only data from t_0_ or t_1_ confirmed the previous baseline analysis. We observed a loss of explained variance in the prospective analysis models. This loss was greatest for job satisfaction, suggesting that this outcome is most influenced by short-term working conditions.

**Conclusions:**

Both the COPSOQ and ERI instruments show good criterion validity and adequately predict contemporaneously measured self-reported measurements of health and (occupational) well-being. However, the COPSOQ provides a more detailed picture of working conditions and might be preferable for improvment strategies in workplaces. Additional prospective research with shorter follow-up times would be beneficial for estimating dose-response relationships.

**Supplementary Information:**

The online version contains supplementary material available at 10.1186/s12889-021-12240-3.

## Background

The World Health Organization (WHO), in its Healthy Workplace Framework and Model [1], states that the healthy workplace should include not only a healthy physical work environment but among others, should comprise healthy psychosocial conditions that protect and promote health and safety. A healthy workplace not only benefits the employee, but also the employer, increasing productivity and minimizing the number of sick days taken.

Two models have been widely used to characterize psychosocial conditions at work, the Demand-Control-Support [2] and the Effort-Reward-Imbalance models [3]. The Demand-Control Support model [4] posits that high demands and low control at the workplace lead to adverse health outcomes. The Effort-Reward-Imbalance Model (ERI) [5] suggests that an imbalance between efforts made and rewards received by the employee also leads to negative health effects. Both models have shown that adverse psychosocial work conditions increase the risk of incident depression [6, 7], stress-related disorders [8], musculoskeletal complaints [9], and incident cardiovascular outcomes [10–16] in prospective studies.

While other psychosocial working conditions, such as role conflict, job uncertainty, mobbing, and conflict at work have also been associated with ill health, absenteeism, and general ability to work [17–19], few studies have considered the health effects of these workplace conditions in prospective studies. Prospective studies find mobbing is associated with incident depression [18] and CVD (Cardiovascular disease) in men [20], and repeated surveys of workers in Finland found ergonomic conditions, opportunities for development, and work organization were associated with workability at follow-up [21, 22]. The systematic review of Aronsson and colleagues [23] summarized evidence from prospective studies of working conditions and burnout, finding evidence of associations between low job control, job demands, and workplace support with burnout, but few prospective studies examining other working conditions. In a prospective study, Burr, et al. [24] determined that age does not modify how a high work pace, low influence at work, and possibilities for development affect self-rated health after five years. Prospective studies examining the impact of various psychosocial working conditions on job satisfaction or satisfaction with life are also lacking. So far, studies have also not tried to determine which forms of psychosocial working conditions out of the many candidates have the greatest influence on future health conditions and well-being.

The Gutenberg Health Study is a large population-based study based in and around the German city of Mainz that began initially with a focus on cardiovascular health. Various occupational, lifestyle, genetic, and environmental factors are measured at regular intervals to determine their effects on a wide array of health outcomes [25]. The psychosocial working situation of the first wave (*n* = 5000) of participants recruited into the GHS cohort at baseline was described previously [26]. This earlier analysis focused on comparing the validity of the ERI questionnaire and the Copenhagen Psychosocial Questionnaire (COPSOQ). In contrast to the ERI questionnaire, the COPSOQ [27] is based on several theoretical models widely used to assess psychosocial conditions at work, including the above-mentioned Demand-Control Support model, the ERI model, and several others, such as the job characteristics model, the Michigan organizational stress model, the socio-technical approach, the action-theoretical approach, and the vitamin model (see also Kompier [28]). In the initial baseline evaluation, the association between psychosocial working conditions and internal outcomes integrated into the standard COPSOQ (i.e. job satisfaction, self-rated health, burnout, and satisfaction with life) were considered to determine the interval validity of the measured criteria for predicting the outcomes measured contemporaneously.

### Aims of the current study

The aims of this current study are i) to replicate the preliminary baseline analysis with the whole GHS sample (*n* = 15,010) surveyed at baseline and at the five-year follow-up, and ii) to prospectively examine psychosocial conditions at work by testing to what extent psychosocial conditions experienced at baseline predict the self-rated (COPSOQ) outcomes (i.e., job satisfaction, general health, burnout, and satisfaction with life) measured after five years. We use no fixed hypotheses that certain a priori defined parameters are relevant, which would lead to a confirmatory model testing. Our analysis is exploratory in the sense that our goal is to find out which of the psychosocial working conditions are included in the models as predictors of the outcomes.

## Methods

### Study participants

Starting in 2007 a total of 15,010 participants were recruited into the GHS cohort. Residents of Mainz and the surrounding area (Mainz-Bingen) between the age of 35 and 74 years were randomly selected (stratified 1:1 by sex and rural vs urban) and invited to participate in the study. People unable to communicate in German or who were physically unable to attend the baseline examinations at the study center were excluded. All participants gave their written informed consent to participation. The Medical Ethics Commission of Rhineland-Palatinate and local and Gutenberg-University of Mainz data protection officials reviewed and approved the study (ethics committee review number 837.020.07(5555)).

Between 2007 and 2012, five-hour baseline examinations were conducted at the University Medical Center of the Johannes Gutenberg University of Mainz [25]. The baseline response among all successfully contacted residents was 70% [29]. Of the participants between the ages of 35 and 64 years and in paid employment at baseline (*n* = 8306), 66 (0.8%) died before the 5-year follow-up and a further 980 (11.9%) were lost to follow-up (C. Drossard, personal communication, March 25, 2021).

Self-ratings of psychosocial working conditions were obtained at baseline (t_0_) among the working population using either the COPSOQ or the Effort-Reward-Imbalance (ERI) questionnaires. At baseline, the distribution of the questionnaires alternated weekly. At the five-year follow-up (t_1_) the subjects still working received the same questionnaire filled out at baseline. The general characteristics of the participants filling out either the COPSOQ or ERI questionnaire at each time point, including information on age, sex, education, and occupational sector, were analyzed descriptively.

### COPSOQ

The COPSOQ is a validated and comprehensive questionnaire used internationally to measure a wide variety of psychosocial working conditions [27]. As Kristensen, et al. [27] write, the COPSOQ is intended to be as broad and comprehensive as possible by covering possibly all relevant psychosocial workplace factors. The COPSOQ is thus, “theory-based without being based on one specific theory” [27]. The questionnaire includes a wide variety of aspects on working conditions taken from different theories and models in order to assess psychosocial working conditions as comprehensively as possible. Approximately half of the GHS participants who were working at baseline were assessed with the German standard version of the COPSOQ that comprised 25 scales in the dimensions: demands, influence and development, support and leadership, further parameters, and outcomes (strain/effects) [26, 30, 31]. The COPSOQ scaled to values between 0 and 100 (minimum and maximum value, respectively). Staying with the standard procedure for COPSOQ, scale values are calculated based on the available answers if at least half of the scale items are answered; if less than half of the items are answered, the scale value is set to missing.

Depending on the content of the scales, higher scores can represent either favorable or unfavorable conditions. Higher scores represent favorable conditions for the scales measuring influence at work, degree of freedom at work, possibilities for development, meaning of work and workplace commitment, predictability, role clarity, quality of leadership, social support, feedback, social relations, and sense of community. In the case of the scales for quantitative demands, emotional demands, demands for hiding emotions, work-privacy conflict, job insecurity, role-conflicts, and mobbing, higher scores represent unfavorable conditions.

The demand domain of the COPSOQ included four scales:

- quantitative demands were estimated with four items (e.g. Do you have to work very fast? always; often; sometimes; seldom; never/hardly ever),
- emotional demands with three items (e.g. Is your work emotionally to a very large extent; to a large extent; somewhat; to a small extent; to a very small extent),
- demands for hiding emotions with two items (e.g. Does your work require that you hide your feelings? to a very large extent; to a large extent; somewhat; to a small extent; to a very small extent), and
- work-privacy-conflict with five items (e.g. The demands of my work interfere with my private and family life. Agree fully; agree somewhat; undecided; disagree somewhat; disagree).

Five scales belonged to the thematic domain of influence and development: influence at work (4 items; e.g. Do you have a large degree of influence on the decisions concerning your work? always; often; sometimes; seldom; never/ hardly ever),

- degree of freedom of work (4 items; e.g. Can you decide when to take a break? always; often; sometimes; seldom; never/hardly ever),
- possibilities for development at work (4 items; Do you have the possibility of learning new things through your work to a very large extent; to a large extent; somewhat; to a small extent; to a very small extent),
- meaning of work (3 items; e.g. Is your work meaningful? to a very large extent; to a large extent; somewhat; to a small extent; to a very small extent), and
- workplace commitment (4 items; Are you proud of being part of this company? to a very large extent; to a large extent; somewhat; to a small extent; to a very small extent).

The interpersonal relations and leadership domain comprised nine scales:

- predictability (2 items; e.g. At your place of work, are you informed well in advance concerning for example, important decisions, changes, or plans for the future? to a very large extent; to a large extent; somewhat; to a small extent; to a very small extent),
- role-clarity (4 items; e.g. Do you know exactly which areas are your responsibility? to a very large extent; to a large extent; somewhat; to a small extent; to a very small extent),
- role conflicts (4 items; e.g. Are contradictory demands placed on you at work? to a very large extent; to a large extent; somewhat; to a small extent; to a very small extent),
- quality of leadership (4 items; e.g. To what extent would you say your immediate superior makes sure that the members of staff have good development opportunities? to a very large extent; to a large extent; somewhat; to a small extent; to a very small extent; I don’t have a superior/colleagues),
- social support (4 items; e.g. How often do you get help and support from your colleagues, if needed? always; often; sometimes; seldom; never/hardly ever; I don’t have a superior/colleagues),
- feedback (2 items; How often does your immediate superior talk with you about how well you carry out your work? always; often; sometimes; seldom; never/hardly ever; I don’t have a superior/colleagues),
- social relations (2 items; e.g. Is it possible for you to talk to your colleagues while you are working? always; often; sometimes; seldom; never/hardly ever; I don’t have a superior/colleagues),
- sense of community (3 items; e.g. Is there a good atmosphere between you and your colleagues at work? always; often; sometimes; seldom; never/hardly ever; I don’t have a superior/colleagues), and
- mobbing (1 item; How often do you feel unjustly criticized, bullied or shown up in front of others by your colleagues and your superior? always; often; sometimes; seldom; never/hardly ever; I don’t have a superior/colleagues).

As a further parameter, job insecurity was measured with 4 items (e.g. Are you worried about becoming unemployed? to a very large extent; to a large extent; somewhat; to a small extent; to a very small extent).

More information on the German standard version of COPSOQ can be found at www.copsoq.de, more information on the international development of the questionnaire at www.copsoq.network.org.

### Effort-reward-imbalance

The ERI questionnaire (2006 version when the survey was started) included 6 effort items, 11 reward items, and 6 items regarding overcommitment. German versions of the ERI questionnaire have been validated and tested in the general population [32, 33]. The ERI-items first asked if a work condition was experienced, and then a follow-up question asked for a rating of the strain related to this working condition. For example, the presence of physical demands at work was assessed with a yes or no response to the statement, “My work is/was physically demanding”. If yes was selected, “And how much does that strain you?” was asked with four answer possibilities (i.e. not at all, moderately, strongly, very strongly). The ERI-ratio was calculated by dividing the sum of the effort items by the reward and correcting for the difference in number of items. Ratio values diverging from 1 indicate an imbalance between effort expended and reward received: values over one indicate the effort experienced exceeded the reward, and values under one indicate the reward experienced exceed the effort. To ease comparison with the COPSOQ items, the ERI results were also rescaled to values between 0 and 100.

### Outcomes

Among both groups of participants, four health and work strain outcomes were also assessed. These outcomes are part of the standard version of the COPSOQ, and we included them into the ERI to enable comparisons between the COPSOQ and ERI instruments and analyze criterion validity [26]. The internal outcomes measured were job satisfaction, general health status, burnout symptoms, and the satisfaction with life scale (SWLS). Job satisfaction was assessed using seven items (e.g. If you look at your work situation as a whole, how satisfied are you with your career prospects?) with four answer possibilities (i.e. very satisfied, satisfied, unsatisfied, or very unsatisfied). General health status was assessed with one question from the EQ-5D (EuroQol) where health was rated on a scale from 0 (worst health imaginable) to 10 (best health imaginable) using the question: “Your state of health: If you rate the best conceivable state of health with 10 points and the worst conceivable with 0 points: How many points would you award your current state of health?”. Burnout was assessed with six questions from the Copenhagen Burnout Inventory (CBI) (e.g. How often do you feel tired? always, often, sometimes rarely, never). Satisfaction with life was assessed with five items from the Satisfaction with Life Scale (SWLS) (e.g. In most areas my life corresponds with my ideal expectations. Agree completely; agree; agree somewhat; neither agree nor disagree; disagree somewhat; disagree; disagree completely) [34]. All of the outcomes were rescaled to values between 0 and 100. Increasing values represent more desirable levels of job satisfaction, general health, and satisfaction with life. However, for the adverse outcome of burnout, rising values are less desirable.

### Statistical analyses

We analyzed the results of the COPSOQ and ERI questionnaires at both study time points descriptively using means and standard deviations. In correlation analyzes, all bivariate relations between workplace factors and outcome factors were assessed. In a final step, linear regression models were selected using stepwise selection to consider the associations between psychosocial working conditions and health or strain outcomes measured contemporaneously. The same method was used in several international COPSOQ validation studies (i.e. [30, 35]) as well as in the previous analyses with the data of the GHS [26]. The prospective relationships between the psychosocial conditions at baseline and the outcomes at follow-up were also analyzed with multiple linear regression models. For each outcome, one COPSOQ model and one ERI model were selected with stepwise selection; models presented always include only the selected predictors, thus maximizing case numbers available.

Model selection for the ERI subgroup included the variables for the rescaled (0–100) effort, reward and overcommitment scales, as well as the ERI ratio. For the COPSOQ linear regression models, we report the model selected in the fifth step of model selection, unless the next model selection step resulted in an improvement in explained variance (R^2^) by two or more percentage points. This was done because the number of factors that are statistically significant is associated with the sample size, which is, in this case quite large. Based on our analyses at baseline, after the first five scales were selected, additional model variables, although statistically significant, explained little additional variance in all of the dependent outcome variables. Therefore, we also stopped the selection of prospective models after five variables were selected. This also ensured the comparability of results with the results reported in the German COPSOQ validation [31] and at baseline [26]. Collinearity of the models was assessed with the variance inflation factor (VIF) using a cut-off of 5. We examined the outcome variance explained by the models (R^2^) to estimate and compare the predictive performance of the questionnaires. To give a complete picture of all relations between (candidate) predictors and outcomes correlation matrixes of all workplace factors with the outcome factors (Pearson correlation coefficients) are shown in the supplementary material (Supplementary Tables S3-S6).

All of the analyses were conducted in SPSS 23. Like the initial baseline study described by Nübling, et al. [26], we did not adjust for age and sex since the age and sex distribution of the groups receiving the ERI or COPSOQ were comparable. In a sensitivity analysis, we adjusted for age, sex, and socio-economic status. The socio-economic status index comprised information on education, income, and occupational status and ranged from 3 to 21, with higher values indicating higher status.

## Results

### Descriptive analysis

Of the 15,010 study participants, 8680 (57.8%) persons currently employed received either the COPSOQ or ERI questionnaire at baseline. The rest of the cohort were excluded from further analysis, because they did not fill out a questionnaire at baseline (e.g. not working, retired) or only completed a questionnaire at the follow-up. In total, 4322 people were included in the COPSOQ sample at baseline, and 71.5% (*n* = 3091) of these participants also filled out a COPSOQ questionnaire at follow-up. The ERI sample comprised 4358 participants at baseline, of which 72.1% (*n* = 3142) filled out a second ERI questionnaire after five years.

Approximately one-third of the sample did not fill out a questionnaire at the five-year follow-up. This was largely due to retirement, which also explained the older age distribution of the participants only included at baseline (Table 1). The average age of the COPSOQ and ERI participants that filled out questionnaires at both time-points was lower than that of the group only assessed at baseline (COPSOQ: 49.0 vs. 53.6 years and ERI: 47.3 years versus 53.2 years). Overall slightly more men (53.9%) than women were included in the analysis. The distribution of men and women in the ERI and COPSOQ groups was similar. Also, there were no distributional differences for COPSOQ and ERI within the occupational sectors. Although the distributions were similar, a higher percentage of participants for whom we could not determine the occupational sector participated solely at baseline. In the groups of participants with questionnaires at both time points, the proportion of men increased by about two percentage points for both questionnaires.

**Table 1:** Baseline demographic data on participants with COPSOQ / ERI

	Total	COPSOQ (t_0_ & t_1_)	COPSOQ (only t_0_)	ERI (t_0_ & t_1_)	ERI (only t_0_)
**Total**	8680 (100%)	3091 (100%)	1231 (100%)	3142 (100%)	1216 (100%)
**Sex**					
Women	3998 (46.1%)	1386 (44.8%)	590 (47.9%)	1446 (46.0%)	576 (47.4%)
Men	4682 (53.9%)	1705 (55.2%)	641 (53.1%)	1696 (54.0%)	640 (52.6%)
**Age** mean (SD)	49.0 (8.2)	47.2 (7.2)	53.6 (8.9)	47.3 (7.2)	53.2 (8.9)
**Age Group** years					
35–44	2858 (32.9%)	1207 (39.0%)	243 (19.7%)	1156 (36.8%)	252 (20.7%)
45–54	3458 (39.8%)	1384 (44.8%)	295 (24.0%)	1465 (46.6%)	314 (25.8%)
55–64	2125 (24.5%)	465 (15.0%)	609 (49.5%)	479 (15.2%)	572 (47.0%)
65–74	239 (2.8%)	35 (1.1%)	84 (6.8%)	42 (1.3%)	78 (6.4%)
**School Education**					
9th grade	2422 (27.9%)	718 (23.2%)	477 (38.8%)	757 (24.2%)	470 (38.7%)
10th grade	2062 (23.8%)	741 (24.0%)	265 (21.5%)	793 (25.2%)	263 (21.7%)
12th/13th grade	4144 (47.4%)	1607 (52.0%)	468 (38.0%)	1571 (50.1%)	468 (38.6%)
Other certification	39 (0.5%)	11 (0.4%)	13 (1.1%)	8 (0.3%)	7 (0.6%)
None	30 (0.4%)	12 (0.4%)	7 (0.6%)	5 (0.2%)	6 (0.5%)
**Occupational Education**					
Vocational school/apprenticeship	3622 (41.7%)	1222 (39.6%)	589 (48.0%)	1267 (40.5%)	544 (45.0%)
Technical school/master craftsman	1356 (15.6%)	511 (16.6%)	173 (14.1%)	484 (15.5%)	188(15.5%)
University / College of applied science	3122 (36.0%)	1198 (38.8%)	366 (29.8%)	1196 (38.2%)	362 (15.5%)
Other qualification	191 (2.2%)	61 (2.0%)	29 (2.4%)	79 (2.5%)	22 (1.8%)
**Occupational Sector**					
Agriculture, forestry, and horticulture	214 (2.5%)	80 (2.6%)	25 (2.0%)	81 (2.6%)	28 (2.3%)
Production of raw materials and goods, and manufacturing	1089 (12.6%)	408 (13.2%)	138 (11.2%)	418 (13.3%)	125 (10.3%)
Construction, architecture, surveying and technical building services	385 (4.4%)	136 (4.4%)	64 (5.2%)	140 (4.5%)	45 (3.7%)
Natural sciences, geography and informatics	535 (6.2%)	231 (7.5%)	50 (4.1%)	201 (6.4%)	53 (4.4%)
Traffic, logistics, safety and security	680 (7.8%)	230 (7.4%)	111 (9.0%)	223 (7.1%)	116 (9.5%)
Commercial services, trading, sales, the hotel business and tourism	792 (9.1%)	271 (8.8%)	123 (10.0%)	285 (9.1%)	113 (9.3%)
Business organization, accounting, law and administration	2368 (27.3%)	871 (28.2%)	297 (24.1%)	881 (28.0%)	319 (26.2%)
Health care, the social sector, teaching and education	1533 (17.7%)	540 (17.5%)	209 (17.0%)	588 (18.7%)	196 (16.1%)
Philology, literature, humanities, social sciences, economics, media, art, culture, and design	390 (4.5%)	148 (4.8%)	44 (3.6%)	149 (4.7%)	49 (4.0%)
Other, no answer/ not classified	664 (7.7%)	176 (5.7%)	170 (13.8%)	176 (5.6%)	172 (14.2%)

### SD standard deviation

The results of the ERI questionnaire and the measured outcomes at baseline and at the five-year follow-up are shown in Table 2. Percentages of missing responses are mostly low (< 5%), with the exception of the reward scale and the ERI-Quotient (23%). This because some reward-scale items contain the response category “I have no supervisor” or “I have no colleagues”, which can’t be used for the calculation of scale values. Effort, reward, and overcommitment remained relatively stable over time, and the ERI-Quotient remained the same at 0.5 (SD 0.23) indicating a low risk of an effort-reward imbalance at both points of measurement. For the most part, the assessed outcomes were also comparable over the two time periods. Job satisfaction, general health, and satisfaction with life scores increased minimally (between 0.5 and 2 points out of 100), while the CBI burnout score decreased by 1.7 points at the five-year follow-up. This small improvement in outcomes might be an indication of a *healthy-worker effect*. People experiencing more strain may be more likely to have left the workforce during the five years.

**Table 2:** Distribution of ERI Items and Outcomes at baseline & follow up

	Baseline (t0)				5-year follow-up (t1)				Mean Difference
	N	Min/Max	Mean	SD	N	Min/Max	Mean	SD	
Effort (6 items)	4221	6/29	13.4	4.3	3047	6/30	13.2	4.3	−0.2
Reward (11 items)	3385	11/55	48.6	6.5	2514	15/55	48.8	6.3	0.2
ERI-Quotient (Effort/Reward)	3336	0.20/2.98	0.5	0.23	2469	0.20/2.81	0.5	0.23	0
Overcommitment (6 Items)	4206	6/24	13.2	3.8	3049	6/24	12.9	3.8	−0.3
Effort (0–100)	4318	0/100	31.0	18.0	3108	0/100	29.9	18.0	−1.1
Reward (0–100)	4261	0/100	86.0	14.7	3085	9.1/100	86.6	14.2	0.6
ERI Quotient (Effort/Reward, 100 scale)	4263	0/856	39.5	34.6	3084	0/779	37.8	34.7	−1.7
Overcommitment (0–100)	4308	0/100	39.9	21.3	3102	0/100	38.3	21.3	−1.6
**Outcomes (0–100)**									
Job satisfaction	4264	4.71/100	67.9	15.4	3090	0/100	69.0	15.5	1.1
General health (1 Item)	4302	0/100	72.5	16.6	3110	10/100	73.0	15.7	0.5
Copenhagen Burnout Inventory	4278	0/100	37.7	17.4	3077	0/100	36.0	18.3	−1.7
Satisfaction with Life Scale	4265	0/100	69.6	18.3	3071	0/100	71.6	17.7	2.0

### SD standard deviation

The distribution of the COPSOQ items and outcomes at baseline and the five-year follow-up are shown in Table 3. For the COPSOQ scales percentages of missing responses are mostly very low (< 2%) despite scales containing items with the response category “I have no supervisor” or “I have no colleagues”, i.e. quality of leadership or social support. Up to ca. 15% of participants reported not having either a supervisor or colleagues, which led to a missing scale value. Out of 19 scales concerning workplace factors and four comcerning outcomes in the COPSOQ, one scale has mean values of 80 points or above indicating ceiling effects (role clarity, 80 points at baseline and follow-up) and one scale has mean values of 20 points or below indicating floor effects (mobbing, 16.1 points at baseline and 15.2 at follow-up).

**Table 3:** Distribution of COPSOQ Scales and Outcomes at baseline & follow up

	Baseline (t0)				5-year follow-up (t1)				Mean Difference
	N	Min/Max	Mean	SD	N	Min/Max	Mean	SD	
Quantitative Demands	4294	0/100	49.4	20.9	3080	0/100	48.2	20.9	−1.2
Emotional Demands	4254	0/100	45.6	22.3	3052	0/100	44.2	22.3	−1.4
Demands for hiding emotions	4267	0/100	35.6	24.7	3053	0/100	34.3	25.2	−1.3
Work-Privacy Conflict	4271	0/100	35.4	27.0	3069	0/100	33.1	26.5	−2.3
Influence at work	4267	0/100	53.5	26.5	3064	0/100	53.6	26.6	0.1
Degree of freedom at work	4277	0/100	67.6	25.2	3068	0/100	69.1	24.8	1.5
Possibilities for development	4274	0/100	71.6	20.0	3064	0/100	72.2	19.3	0.6
Meaning of work	4273	0/100	76.5	18.3	3064	0/100	76.6	18.3	0.1
Workplace commitment	4267	0/100	61.0	20.5	3060	0/100	59.8	20.3	−1.2
Predictability	4219	0/100	63.5	21.9	3030	0/100	63.8	21.6	0.3
Role clarity	4254	0/100	80.0	16.0	3043	0/100	80.0	16.1	0
Role conflicts	4245	0/100	38.6	20.0	3037	0/100	37.5	19.9	−1.1
Quality of leadership	3420	0/100	52.2	22.7	2459	0/100	51.7	23.0	−0.5
Social Support	3776	0/100	65.8	20.2	2727	0/100	66.4	19.6	0.6
Feedback	3788	0/100	45.3	22.5	2723	0/100	44.3	22.3	− 1.0
Social relations	3747	0/100	55.8	28.8	2721	0/100	56.8	28.7	1.0
Sense of community	3737	0/100	79.3	16.4	2715	0/100	79.1	16.2	−0.2
Mobbing (1-item)	3679	0/100	16.1	20.8	2663	0/100	15.2	20.6	−0.9
Job insecurity	3548	0/100	24.3	20.5	2538	0/100	21.4	20.1	−2.9
**Outcomes (0–100)**									
Job satisfaction	4216	0/100	69.1	15.0	3029	0/100	70.0	15.0	0.9
General health (1 Item)	4217	0/100	72.8	16.6	3028	0/100	73.3	15.5	0.5
Copenhagen Burnout Inventory	4272	0/100	37.8	17.7	3067	0/100	35.5	18.2	−2.3
Satisfaction with Life Scale	4261	0/100	70.5	17.9	3064	0/100	72.1	17.2	1.6

Within the COPSOQ sample above, improvements were also observed over time with demand scales decreased by 1.2 to 2.3 points out of 100 on average. The largest decrease in demands was observed for work-privacy conflict, which may be due to the aging of the cohort and the possible reduction in private, family-related obligations over time.

Degree of freedom at work increased by the five-year follow-up (1.5/100) and workplace commitment decreased by 1.2/100 on average, but the influence short-scale remained mostly the same. Role clarity did not change, but the average role conflicts score decreased by 1.1/100. While the average social relations score (1.0/100) and the social support scores (0.6/100) increased slightly, the sense of community score (−0.2/100) decreased at the follow-up.

Altogether the largest difference observed by follow-up was for job insecurity. The average job insecurity score decreased by 2.9 out of 100 points by the five-year follow-up. This may reflect the decreasing rates of unemployment experienced in Germany over this time period [36].

As with the ERI sample, the outcomes indicated an improvement in self-rated health and symptoms of strain over time. Most notably, a decrease in the CBI of 2.3/100 on average indicated less indication of burnout symptoms after five years. The change in general self-rated health was the same as the ERI sample (0.5/100), and the changes in job satisfaction (0.9/100) and in the satisfaction with life score (1.6/100) were comparable to that of the ERI sample in Table 2.

### Analyses concerning ERI

The reward scale was most strongly associated with the outcomes in the cross-sectional analysis (Table 4). As expected, the experienced reward was positively associated with job satisfaction, general health, and SWLS, and negatively associated with burnout.. Job satisfaction was best predicted by the ERI model scales, with over 40% of the variance explained by ERI scales at baseline. In contrast, the selected models only explained 10% of the observed variance in the general health outcome. Cross-sectional model selection at follow-up resulted in different models, but a similar amount of variance was explained by all four models (Supplementary Table S1).

**Table 4:** ERI cross-sectional linear regression models selected with forward stepwise regression (factors shown in the order of selection) for four health and work related outcomes at the baseline (t0)

	ERI (t0)		
**Outcomes at t0**	**Scales**	**Beta (SE)**	**R**^**2**^
**Job satisfaction**	Reward	0.689 (0.016)	0.43
	Effort	−0.069 (0.018)	
	Overcommitment	−0.047 (0.010)	
	Ratio	0.035 (0.010)	
	*N* = 4217		
**General health**	Reward	0.248 (0.017)	0.10
	Overcommitment	−0.127 (0.012)	
	*N* = 4228		
**Burnout**	Reward	−0.333 (0.021)	0.27
	Overcommitment E	0.242 (0.013)	
	Effort	0.159 (0.023)	
	Ratio N	−0.033 (0.013)	
	*N* = 4200		
**Satisfaction with Life Scale**	Reward	0.496 (0.022)	0.19
	Overcommitment	−0.134 (0.013)	
	Ratio	0.026 (0.010)	
	*N* = 4191		

*R*^*2*^ *= Proportion of the variance explained by the model; SE Standard Error; N valid case number (fluctuates due to missing values)*

Prospectively, the ERI scales at baseline explained less of the variance in the outcomes measured after five years, with the R^2^ of the prospective models ranging between 0.07 and 0.19 (Table 5). As with the cross-sectional analyses, general health was poorly predicted by the ERI scales, but the R^2^ was not much lower compared to the cross-sectional models. Reward, overcommitment, and the ERI ratio measured at baseline explained about five percentage points less variance for SWLS measured at follow-up. The reward scale was also most strongly associated with the outcomes in the prospective analysis.

**Table 5:** ERI prospective linear regression models selected with forward stepwise regression (factors shown in the order of selection). Four health and work-related outcomes at the 5-year follow–up (t1) were predicted prospectively by the ERI values at baseline (t0)

	ERI (t0)			ERI (t0) adjusted for t0 values of outcomes		
Outcomes at t1	Scales	Beta (SE)	R^**2**^	Scales	Beta (SE)	R^**2**^
**Job satisfaction at t1**	Reward	0.447 (0.018)	0.19	Reward	0.142 (0.022)	0.31
	Overcommitment	−0.049 (0.013)		Overcommitment	− 0.027 (0.012)	
	*N* = 3046			*N* = 3034		
**General health at t1**	Reward	0.206 (0.020)	0.07	Reward	0.087 (0.018)	0.29
	Overcommitment	−0.099 (0.014)		Overcommitment	−0.041 (0.012)	
	*N* = 3057			*N* = 3049		
**Burnout**	Reward	−0.292 (0.023)	0.15	Reward	−0.095 (0.018)	0.46
**at t1**	Overcommitment E	0.190 (0.018)				
	Effort	0.064 (0.021)				
	N					
	*N* = 3027			*N* = 3002		
**Satisfaction with Life Scale**	Reward	0.442 (0.026)	0.14	Reward	0.107 (0.018)	0.50
**at t1**	Overcommitment	−0.111 (0.016)				
	Ratio	0.035 (0.013)				
	*N* = 3022			*N* = 2993		

*R*^*2*^ *= Proportion of the variance explained by the model; SE Standard Error; N valid case number (fluctuates due to missing values)*

Adjusting for baseline levels of the outcome should provide a description of the “isolated” five-year effect of psychosocial conditions on the outcomes. After this adjustment, the reward scale was still most strongly associated with the outcomes measured after five years, but the regression coefficients (Beta) sank considerably. Further adjustment for age, sex, and socioeconomic stuatus had little effect on the regression coefficients (Supplementary Tables S7 and S8).

### Analyses concerning COPSOQ

The fifth linear regression model selected with forward stepwise selection for the cross-sectional analysis of the COPSOQ scales is shown in Table 6. The outcome variance explained by the COPSOQ models were generally comparable to values observed from the cross-sectional ERI models for the same outcomes. Job satisfaction was also best predicted, with around 50% of the observed variance explained by the five COPSOQ scales selected. Cross-sectional models for the five-year follow-up are shown in the on-line appendix (Supplementary Table S2). Although the model performance remained constant at both assessment time points, the selected scales sometimes varied between baseline and follow-up. Only the scales for quality of leadership, meaning of work, and sense of community were consistently positively associated with job satisfaction.

**Table 6:** COPSOQ cross-sectional linear regression models selected with forward stepwise regression for four health and work related outcomes at the baseline (t0) (factors shown in the order of selection)

	COPSOQ (t0)		
Outcomes at t0	Scales	Beta (SE)	R^**2**^
**Job satisfaction**			0.52
	Quality of leadership	0.201 (0.009)	
	Meaning of work	0.239 (0.011)	
	Sense of community	0.204 (0.012)	
	Degree of freedom at work	0.078 (0.007)	
	Job Insecurity	−0.097 (0.009)	
	*N* = 3164		
**General health**			0.11
	Job insecurity	−0.118 (0.015)	
	Possibilities for development	0.165 (0.016)	
	Emotional demands	−0.078 (0.015)	
	Work-Privacy conflict	−0.061 (0.012)	
	Mobbing (1 Item)	−0.069 (0.015)	
	*N* = 3151		
**Burnout**			0.31
	Work-Privacy conflict	0.193 (0.011)	
	Possibilities for development	−0.215 (0.015)	
	Emotional demands	0.187 (0.014)	
	Job insecurity	0.132 (0.014)	
	Mobbing (1 Item)	0.105 (0.013)	
	N = 3164		
**Satisfaction with Life**			0.21
**Scale**	Job insecurity	−0.173 (0.014)	
	Meaning of work	0.095 (0.020)	
	Work-Privacy conflict	−0.121 (0.011)	
	Possibilities for development	0.170 (0.018)	
	Sense of community	0.139(0.019)	
	*N* = 3159		

*R*^*2*^ *= Proportion of the variance explained by the model. SE Standard Error; N valid case number (fluctuates due to missing values)*

The prospective prediction of the outcomes with the COPSOQ scales also performed similarly to the selected ERI models (Table 7). Without adjusting for the baseline outcome values, the outcome variance explained by baseline COPSOQ scales in the prospective analysis was about half of that observed in the cross-sectional analysis. Also here, the baseline outcome values were strongly associated with the follow-up outcome levels, and the strength of these associations resulted in fewer scales being selected in the final models. The model coefficients also were greatly attenuated by inclusion of the baseline outcome values. Adjustment the final models for age, sex, and socio-economic status had no effect on the direction of the regression coefficients, and little effect on the magnitude of the coefficients (Supplementary Tables S9 and S10).

**Table 7:** COPSOQ prospective linear regression models selected with forward stepwise regression (factors shown in the order of selection). Four health and work related outcomes at the 5-year follow–up (t1) were predicted by the COPSOQ scales at baseline (t0)

	COPSOQ (t0)			COPSOQ (t0) adjusted for t0 values of outcomes		
Outcomes at t1	Scales	Beta (SE)	R^**2**^	Scales	Beta (SE)	R^**2**^
**Job satisfaction at t1**			0.24			0.27
	Meaning of work	0.145 (0.017)		Job insecurity	−0.088 (0.014)	
	Sense of community	0.157 (0.019)		Meaning of work	0.082 (0.018)	
	Predictability	0.098 (0.018)		Sense of community	0.092 (0.019)	
	Job insecurity	−0.110 (0.014)		Predictability	0.070 (0.016)	
	Quality of leadership	0.080 (0.015)				
	*N* = 2281			*N* = 2274		
**General health at t1**			0.07			0.26
	Job insecurity	−0.101 (0.017)		Mobbing (1 Item)	−0.044 (0.015)	
	Mobbing (1 Item)	−0.068 (0.017)		Job insecurity	−0.047 (0.015)	
	Work-Privacy conflict	−0.049 (0.014)		Role conflict	−0.039 (0.016)	
	Possibilities for development	0.091 (0.019)				
	Emotional demands	−0.047 (0.017)				
	*N* = 2270			*N* = 2252		
**Burnout**			0.17			0.47
**at t1**	Work-Privacy conflict	0.113 (0.015)		Job insecurity	0.055 (0.015)	
	Job insecurity	0.151 (0.018)		Demands for hiding emotions	0.029 (0.012)	
	Possibilities for development	−0.168 (0.021)		Possibilities for development	−0.034 (0.016)	
	Emotional demands	0.172 (0.019)				
	Predictability	−0.091 (0.020)				
	*N* = 2296			*N* = 2292		
**Satisfaction with Life**			0.16			0.47
**Scale at t1**	Meaning of work	0.114 (0.023)		Meaning of work	0.056 (0.016)	
	Job insecurity	−0.152 (0.017)		Job insecurity	−0.046 (0.014)	
	Sense of community	0.136 (0.22)		Sense of community	0.061 (0.018)	
	Work-Privacy conflict	−0.077 (0.013)		Social relations	−0.032 (0.009)	
	Possibilities for development	0.114 (0.022)				
	*N* = 2295			*N* = 2287		

*R*^*2*^ *= Proportion of the variance explained by the model; SE Standard Error; N valid case number (fluctuates due to missing values)*

## Discussion

The COPSOQ and ERI are widely used instruments for the evaluation of psychosocial work factors; they assess a wide variety of possible sources of stress (workplace factors) and can be adapted to different occupational branches and changing working conditions. The main objective of this analysis was to assess the criterion validity of both instruments analyzing the relationship of a variety of psychosocial workplace factors to the four concurrent and future outcome factors: job satisfaction, general health, burnout-symptoms (Copenhagen Burnout Inventory, CBI), and satisfaction with life.

The results show, that working conditions measured by COPSOQ and ERI are related to the four outcomes parameters in correlation and regression analyses). We demonstrated this previously for the first 5000 GHS participants recruited at baseline [26], and confirm this now at both baseline and follow-up, based on the entire cohort (N = 15,010). The results presented in this paper for the complete cohort sample were quite similar to the results of the first wave of study participants.

Considering the longitudinal relationship linking baseline stress factors to outcome factors after five years, the amount of variance explained is markedly lower than when linking outcomes and presumed predictors at one time point. This was especially true for job satisfaction for both the ERI and COPSOQ models, where the variance explained with the longitudinal assessment was reduced to less than half of variance explained by the cross-sectional models. For ERI, the “prospective” R^2^ was 0.19 versus the “cross-sectional” R^2^ of 0.43 at baseline and 0.42 at follow-up. Similarly, the R^2^ was 0.24 for the prospective COPSOQ model and 0.52 and 0.56 for the two cross-sectional models, respectively. The reduction of explained variance was not as pronounced for the prospective models of the other three outcomes, and this was especially true for satisfaction with life. One possible explanation for this finding might be that job satisfaction is a more temporary and unstable outcome that is probably influenced more by short-term (working) conditions than satisfaction with life. Thus, current workplace factors may have a less stable influence on future job satisfaction than on future satisfaction with life after five years.

We observed a reduction of the variance explained in the prospective analysis. In part this is probably due to the artefact of predictive power overestimation caused by common method bias in cross-sectional studies, and thus in the models analyzing one-time point only (baseline or follow-up). Common method bias is reduced in longitudinal studies. On the other hand, the loss of explained variance in the prospective models can somehow be expected, as the predictor is only a single exposure measurement point for stress factors that took place five years before. We do not know if the exposure measured at baseline continued for the entire five years until follow up or if exposure factors vanished or changed in the meanwhile. Changes in exposure would explain why contemporaneously measured workplace factors show a stronger relation to the outcomes. A cumulative dose of exposure would be a more accurate description of workplace conditions over time, but such a cumulative dose is hard or almost impossible to achieve.

The systematic review by Aronsson, et al. [23] on working conditions and their relationship to burnout and components of burnout (emotional exhaustion) found that the prospective studies (including case-control studies) most frequently considered the separate components of job strain (i.e., demand and control) with the emotional exhaustion dimension of burnout. Fewer prospective studies considered the relationship between the more specific dimensions of psychosocial working conditions measured by the COPSOQ and burnout. Aronsson, et al. [23] found limited to moderate trustworthiness of evidence for increased risk of emotional exhaustion (a core component of burnout) due to low job control, demands (unspecified and emotional), low social support (from supervisors and co-worker), high workload, low reward, job insecurity, and with lack of workplace justice. We also found that the reward component of the ERI scale was negatively associated with burnout symptoms at follow-up in the ERI subsample after adjusting for the baseline levels of burnout symptoms. In the COPSOQ subsample, job insecurity and job demands for hiding emotions (emotional demands) correlated positively with later burnout symptoms; possibilities for development correlated negatively with later burnout symptoms. In contrast to the meta-analysis by Aronsson, et al. [23], which found the largest Odds Ratio (OR) for risk of emotional exhaustion in association with high workload (OR = 4.22; 95% confidence interval 3.50–5.11; 7 studies), quantitative demands (a comparable COPSOQ scale) was not selected as a predictor of burnout in our analysis.

Prospective studies have considered exposure to psychosocial working conditions and health. Lang, et al. [9] conducted a meta-analysis of prospective studies of psychosocial working conditions and musculoskeletal complaints. Their meta-analysis finds statistically significant increased risks for musculoskeletal complaints with low social support (RR for low back pain = 1.42; 95% 1.25–1.61), high job strain (RR for neck and shoulder complaints = 1.33; 95% CI 1.08–1.62), and highly monotonous work (RR for arm, wrist, and upper extremity symptoms = 1.57; 1.27–1.93). Meta-analyses of prospective studies find an increased risk of depression due to job strain [37] and ERI [38]. A meta-analysis of psychosocial working conditions and mortality found statistically significant increased hazard ratios for all-cause mortality and coronary heart disease mortality due to low job control [39]. The same systematic review found no increased mortality risk for high job demands, job strain, shift work, or job insecurity. An individual-participant data meta-analysis of 11 European cohort studies and ERI and job strain found low reward increased the risk of heart disease [40]. The effort component of ERI, on the other hand, was not associated with an increased risk of coronary heart disease. Similarly, we found the reward component of ERI was beneficial, and the over-commitment component was detrimental for self-reported health after five years. The effort component was not selected as a prospective predictor of general health. In contrast to other prospective research of health outcomes, we found mobbing, job insecurity, and role conflict were strongly associated with poorer self-reported general health at follow-up.

One study with similarities to ours is the prospective population-based study conducted by Burr, et al. [41] in Denmark. Burr, et al. [41] evaluated how COPSOQ dimensions can be used in addition to the ERI or job strain to improve the prediction of self-reported vitality and mental health after five years. Using a similar method of adjusting for baseline values of the outcome, they found that COPSOQ (due to its broader content) was better suited to predict mental health than ERI after the five years. They also postulated that stronger effects might have been detected with a shorter follow-up time. While the ERI model or theory assesses a potential relevant source of workplace strain, strain is also attributable to other working conditions that the ERI model does not measure. This can have practical implications, as both can be used to conduct mandatory psychosocial risk assessments of workplaces in Germany. Using ERI alone may overlook potential sources of preventable strain (i.e. emotional demands are not included in ERI). Since ERI items are more generally formulated, it might also be more difficult to identify concrete critical workplace factors that require improvement.

As in the initial assessment of the first 5000 participants, the outcome job satisfaction remained the outcome most closely related to psychosocial workplace factors for both the COPSOQ and the ERI subsamples. For the smaller 5000-sample the R^2^ was 0.46 with the ERI models [26], and now based on the entire cohort we found an R^2^ of 0.43. Using the COPSOQ models, the R^2^ was somewhat higher for both the 5000-sample (R^2^ = 0.51) and the entire cohort (R^2^ = 0.52). The variance of burnout symptoms explained by the models also remained consistent at R^2^ = 0.26 and 0.27 for the ERI model and at R^2^ = 0.35 and 0.31 for the COPSOQ models. The models of both questionnaires explained about 20% of the variance in the satisfaction with life outcome, and about 10% of the general health variance was explained for both samples. Although the list and ranking of the selected predictors sometimes changed in the models between the first baseline wave, the complete baseline, and follow-up samples, the R^2^-coefficients of the models remained about the same. This indicates that the outcomes are consistently explained at approximately the same level by the psychosocial workplace factors in all samples used.

### Strengths and limitations

The strengths of this study are that it used a large population-based prospective study to consider the long-term effects of psychosocial working conditions. The initial recruitment of the cohort achieved an acceptable response recruitment efficacy of 55.5% (percent of selected persons contacted) and a 70.0% cooperation proportion (percent of contated persons taking part) [29], which should minimize selection bias and improve the representativeness of the GHS cohort.

While the GHS cohort was large, splitting the working population at baseline into ERI and a COPSOQ sub-groups meant that we could only ever consider about half of GHS cohort. While using both the ERI and the COPSOQ gave us the unique opportunity to compare both evaluation tools in the same overall population, we could not directly compare ERI and COPSOQ results in the same study participants. We also cannot see if dimensions of COPSOQ could complement ERI assessments, in order to better predict health outcomes, as Burr, et al. [41] did. While the prospective nature of the study is a major strength of this research, Burr, et al. [41] also used a five-year follow-up and suggested that associations between psychosocial working conditions and self-reported health may have weakened after so much time. Unfortunately, we do not have any information on changes to the self-reported outcomes or the psychosocial working conditions in the years between the baseline and follow-up assessments. Also, we do not know if poor psychosocial working conditions at baseline may have caused some workers to leave the work-force early and retire.

The COPSOQ is a unique evaluation of psychosocial work factors because it assesses a wide variety of possible sources of stress and can be adapted to different occupational branches and changing working conditions. This study shows that working conditions measured by COPSOQ are related to the outcomes job satisfaction, general health, burnout, and satisfaction with life. This criterion validity was shown for the time points at baseline and at follow-up, as well as - to a lesser extent - for the longitudinal relationship where baseline stress factors were related to follow-up outcome factors. Albeit the small beta coefficients observed for the longitudinal models, especially after adjustment for the baseline levels of the self-reported outcomes, shows that predictive information is limited after five years.

The adaptability of the COPSOQ evaluation tool is especially advantageous for assessing contemporary workplace conditions. Workplaces and workplace stress factors are changing, and occupational sciences are developing; thus new factors have to be included in questionnaires of psychosocial factors in order to be up-to-date and to encompass all relevant sources and forms of workplace stress. Since 2013 the “COPSOQ international network” (www.copsoq-network.org) is coordinating the further development of the instrument [42]. Most recently, the new factors like “trust and justice / social capital”, “delimitation of work (temporal and local)”, “inability to relax”, “presenteesm”, and “work engagement” have been included in the COPSOQ. These constructs are already included in the questionnaire being used for the 10-year follow-up of the GHS and can be analyzed in the future.

As the digital revolution progresses, the impact of changes to the workplace due to new technologies and digitalization on employees will also need to be considered. An instrument to assess the health effects of work in digitized and computerized workplaces that could be incorporated into future assessments with COPSOQ has already been developed as part of the Healthy Work in Pioneer Branches study (“Gesunde Arbeit in Pionierbranchen”, GAP) [43]. Future research with the GAP instrument could help determine which changing working conditions related to the digital revolution are salutogenetic or detrimental to health. The GAP-module will also be incorporated into future follow-ups of the GHS.

## Conclusions

Both the COPSOQ and ERI instruments performed comparably well, regarding their psychometric properties, in both the cross-sectional and prospective analyses. However, the COPSOQ instrument provided a more detailed picture of working conditions, and is a flexible assessment tool that can be adapted to changing working conditions. Although providing a long-term assessment of working conditions, the two separate assessments points at a distance of five years can only provide a rough depiction of the development of stress factors over the five-year period since they do not reflect cumulative stress, as would be desirable for epidemiological dose-response analyses. The long-term influence of psychosocial factors at work on outcome factors concerning satisfaction and self-rated health could be statistically proven.

However, the limited longitudinal predictive power we observed was not wholly unexpected from this two-point measurement with questionnaires since this is a quite rough assessment. A more accurate but also much more resource-consuming method would be a complete retrospective or prospective assessment of workplace factors over time, allowing the calculation of cumulative doses of psychosocial exposures and the analyses of dose-response relationships.

## Supplementary Information

**Additional file 1: Table S1**. ERI cross-sectional linear regression models selected with forward stepwise regression (factors shown in the order of selection) for four health and work related outcomes at the baseline (t0) or at the 5-year follow-up (t1). **Table S2**. COPSOQ cross-sectional linear regression models selected with forward stepwise regression (factors shown in the order of selection) for four health and work-related outcomes at the baseline (t0) or at the 5-year follow-up (t1). **Table S3**. Correlation matrix (Pearson correlation coefficients) for COPSOQ dimensions, outcomes (shaded in gray), age and sex at baseline. **Table S4**. Correlation matrix (Pearson correlation coefficients) for COPSOQ dimensions and outcomes (shaded in gray) between baseline and follow-up. **Table S5**. Correlation matrix (Pearson correlation coefficients) for ERI dimensions, outcomes (shaded in gray), age and sex at baseline. **Table S6**. Correlation matrix (Pearson correlation coefficients) for between ERI dimensions at baseline and follow-up. **Table S7**. ERI cross-sectional multivariable linear regression models (factors shown in the order of selection), and adjusted for age, sex, and socioeconomic status. Due to the model selection procedure, the models selected at t0 sometimes differ from the models selected at t1. **Table S8**. ERI prospective linear multivariable regression models (factors shown in the order of selection), and adjusted for age, sex, and socioeconomic status. **Table S9**. COPSOQ cross-sectional multivariable linear regression models (factors shown in the order of selection), and adjusted for age, sex, and socioeconomic status. **Table S10**. COPSOQ prospective linear multivariable regression models (factors shown in the order of selection), and adjusted for age, sex, and socioeconomic status.

## Data Availability

The data that support the findings of this study are available from the management board of the GHS but restrictions apply to the availability of these data, which were used under license for the current study, and so are not publicly available. Data are however available from the authors upon reasonable request and with permission of GHS.

## Abbreviations

CBI: Copenhagen Burnout Inventory
COPSOQ: Copenhagen Psychosocial Questionnaire
ERI: Effort-Reward Imbalance
GAP: Healthy Work in Pioneer Branches “Gesunde Arbeit in Pionierbranchen”
GHS: Gutenberg Health Study
SWLS: Satisfaction with Life Scale
t_0_: Baseline
t_1_: 5-year Follow Up
WHO: World Health Organization
