# Supplementary material for: The Gutenberg health study: a five-year prospective analysis of psychosocial working conditions using COPSOQ (Copenhagen psychosocial Questoinnaire) and ERI (effort-reward imbalance)
Source: BMC Public Health. 2022 Jan 6;22:24. doi: 10.1186/s12889-021-12240-3 (PMC8740453; doi:10.1186/s12889-021-12240-3)
Supplement: Supplementary file 1 — Additional file 1: Table S1. ERI cross-sectional linear regression models selected with forward stepwise regression (factors shown in the order of selection) for four health and work related outcomes at the baseline (t0) or at the 5-year follow-up (t1). Table S2. COPSOQ cross-sectional linear regression models selected with forward stepwise regression (factors shown in the order of selection) for four health and work-related outcomes at the baseline (t0) or at the 5-year follow-up (t1). Table S3. Correlation matrix (Pearson correlation coefficients) for COPSOQ dimensions, outcomes (shaded in gray), age and sex at baseline. Table S4. Correlation matrix (Pearson correlation coefficients) for COPSOQ dimensions and outcomes (shaded in gray) between baseline and follow-up. Table S5. Correlation matrix (Pearson correlation coefficients) for ERI dimensions, outcomes (shaded in gray), age and sex at baseline. Table S6. Correlation matrix (Pearson correlation coefficients) for between ERI dimensions at baseline and follow-up. Table S7. ERI cross-sectional multivariable linear regression models (factors shown in the order of selection), and adjusted for age, sex, and socioeconomic status. Due to the model selection procedure, the models selected at t0 sometimes differ from the models selected at t1. Table S8. ERI prospective linear multivariable regression models (factors shown in the order of selection), and adjusted for age, sex, and socioeconomic status. Table S9. COPSOQ cross-sectional multivariable linear regression models (factors shown in the order of selection), and adjusted for age, sex, and socioeconomic status. Table S10. COPSOQ prospective linear multivariable regression models (factors shown in the order of selection), and adjusted for age, sex, and socioeconomic status. [file 12889_2021_12240_MOESM1_ESM.docx]

**Table S1** ERI cross-sectional linear regression models selected with forward stepwise regression (factors shown in the order of selection) for four health and work related outcomes at the baseline (t0) or at the 5-year follow-up (t1).

|  | **ERI (t0)** |  |  | **ERI (t1)** |  |  |
| --- | --- | --- | --- | --- | --- | --- |
| **Outcomes at t0 or t1** | **Scales** | **Beta (SE)** | **R^2^** | **Scales** | **Beta (SE)** | **R^2^** |
| **Job satisfaction** | Reward | 0.689 (0.016) | 0.43 | Reward | 0.677 (0.016) | 0.42 |
| **at t0 or t1** | Effort | −0.069 (0.018) |  | Overcommitment | -0.062 (0.011) |  |
|  | Overcommitment | -0.047 (0.010) |  |  |  |  |
|  | Ratio | 0.035 (0.010) |  |  |  |  |
|  | N=4217 |  |  | N=3065 |  |  |
| **General health** | Reward | 0.248 (0.017) | 0.10 | Reward | 0.235 (0.020) | 0.10 |
| **at t0 or t1** | Overcommitment | -0.127 (0.012) |  | Overcommitment | -0.123 (0.013) |  |
|  | N=4228 |  |  | N=3069 |  |  |
| **Burnout** | Reward | -0.333 (0.021) | 0.27 | Reward | -0.372 (0.021) | 0.29 |
|  | Overcommitment  E | 0.242 (0.013) |  | Overcommitment  E | 0.257 (0.016) |  |
| **at t0 or t1** | Effort | 0.159 (0.023) |  | Effort | 0.112 (0.019) |  |
|  | Ratio  N | -0.033 (0.013) |  | N |  |  |
|  | N=4200 |  |  | N=3042 |  |  |
| **Satisfaction with Life Scale** | Reward | 0.496 (0.022) | 0.19 | Reward | 0.536 (0.025) | 0.20 |
| **at t0 or t1** | Overcommitment | -0.134 (0.013) |  | Overcommitment | -0.113 (0.015) |  |
|  | Ratio | 0.026 (0.010) |  | Ratio | 0.032 (0.011) |  |
|  | N=4191 |  |  | N=3035 |  |  |

*R^2^ = Proportion of the variance explained by the model; SE Standard Error; N valid case number (fluctuates due to missing values)*

**Table S2** COPSOQ cross-sectional linear regression models selected with forward stepwise regression (factors shown in the order of selection) for four health and work-related outcomes at the baseline (t0) or at the 5-year follow-up (t1).

|  | **COPSOQ (t0)** | | | **COPSOQ (t1)** | | |  |
| --- | --- | --- | --- | --- | --- | --- | --- |
| **Outcomes at t0 or t1** | **Scales** | **Beta (SE)** | **R^2^** | **Scales** | **Beta (SE)** | **R^2^** | |
| **Job satisfaction** |  |  | 0.52 |  |  | 0.56 | |
| **at t0 or t1** | Quality of leadership | 0.201 (0.009) |  | Quality of leadership | 0.177 (0.011) |  | |
|  | Meaning of work | 0.239 (0.011) |  | Meaning of work | 0.227 (0.013) |  | |
|  | Sense of community | 0.204 (0.012) |  | Sense of community | 0.194 (0.014) |  | |
|  | Degree of freedom at work | 0.078 (0.007) |  | Predictability | 0.134 (0.013) |  | |
|  | Job Insecurity | -0.097 (0.009) |  | Work-Privacy conflict | -0.071 (0.008) |  | |
|  | N=3164 |  |  | N=2309 |  |  | |
| **General health** |  |  | 0.11 |  |  | 0.10 | |
| **at t0 or t1** | Job insecurity | -0.118 (0.015) |  | Predictability | 0.058 (0.018) |  | |
|  | Possibilities for development | 0.165 (0.016) |  | Work-Privacy conflict | -0.099 (0.013) |  | |
|  | Emotional demands | -0.078 (0.015) |  | Possibilities for development | 0.097 (0.019) |  | |
|  | Work-Privacy conflict | -0.061 (0.012) |  | Sense of community | 0.082 (0.021) |  | |
|  | Mobbing (1 Item) | -0.069 (0.015) |  | Job insecurity | -0.058 (0.016) |  | |
|  | N=3151 |  |  | N=2299 |  |  | |
| **Copenhagen Burnout** |  |  | 0.31 |  |  | 0.29 | |
| **Inventory** | Work-Privacy conflict | 0.193 (0.011) |  | Work-Privacy conflict | 0.197 (0.014) |  | |
| **at t0 or t1** | Possibilities for development | -0.215 (0.015) |  | Job insecurity | 0.158 (0.017) |  | |
|  | Emotional demands | 0.187 (0.014) |  | Possibilities for development | -0.195 (0.019) |  | |
|  | Job insecurity | 0.132 (0.014) |  | Emotional demands | 0.174 (0.017) |  | |
|  | Mobbing (1 Item) | 0.105 (0.013) |  | Sense of community | -0.122 (0.021) |  | |
|  | N=3164 |  |  | N=2311 |  |  | |
| **Satisfaction with Life** |  |  | 0.21 |  |  | 0.21 | |
| **Scale** | Job insecurity | -0.173 (0.014) |  | Meaning of work | 0.144 (0.021) |  | |
| **at t0 or t1** | Meaning of work | 0.095 (0.020) |  | Job insecurity | -0.134 (0.017) |  | |
|  | Work-Privacy conflict | -0.121 (0.011) |  | Work-Privacy Conflict | -0.114 (0.013) |  | |
|  | Possibilities for development | 0.170 (0.018) |  | Sense of community | 0.124 (0.021) |  | |
|  | Sense of community | 0.139(0.019) |  | Possibilities for development | 0.116 (0.020) |  | |
|  | N=3159 |  |  | N=2308 |  |  | |

*R^2^ = Proportion of the variance explained by the model. SE Standard Error; N valid case number (fluctuates due to missing values)*

**Table S3** Correlation matrix (Pearson correlation coefficients) for COPSOQ dimensions, outcomes (shaded in gray), age and sex at baseline

|  | Quantitative Demands | Emotional Demands | Demands for hiding (emotions | Work-privacy conflict | Influence at work | Degree of freedom at work | Possibilities for development | Meaning of work | Workplace commitment | Predictability | Role clarity | Role conflicts | Quality of Leadership | Social Support | Feedback | Social relations | Sense of community | Mobbing (1 Item) | Job insecurity | Job satisfaction | General health (1 Item) | Copenhagen Burnout Inventory | Satisfaction with Life Scale | Age | Sex | SES |
| --- | --- | --- | --- | --- | --- | --- | --- | --- | --- | --- | --- | --- | --- | --- | --- | --- | --- | --- | --- | --- | --- | --- | --- | --- | --- | --- |
|  | **1** | **2** | **3** | **4** | **5** | **6** | **7** | **8** | **9** | **10** | **11** | **12** | **13** | **14** | **15** | **16** | **17** | **18** | **19** | **20** | **21** | **22** | **23** | **24** | **25** | **26** |
|  | 1 | 0.46 ** | 0.32 ** | 0.60 ** | 0.02 | -0.04 * | 0.31 ** | 0.09 ** | 0.13 ** | -0.06 ** | -0.02 | 0.30 ** | -0.17 ** | -0.13 ** | -0.03 | -0.06 ** | -0.12 ** | 0.14 ** | 0.04 ** | -0.10 ** | -0.06 ** | 0.24 ** | -0.03 * | -0.14 ** | -0.11 ** | 0.30 ** |
|  | 0.46 ** | 1 | 0.44** | 0.47 ** | 0.13 | -0.13  ** | 0.34 | 0.16 | 0.24 ** | 0.01 | 0.01 | 0.28 ** | -0.10 ** | -0.10 ** | 0.02 | -0.14 ** | -0.14 ** | 0.16 ** | 0.00 | -0.07 ** | -0.10 ** | 0.30 ** | -0.05 ** | 0.02 | -0.02 | 0.28 ** |
|  | 0.32 ** | 0.44 ** | 1 | 0.36 ** | -0.11 ** | -0.21 ** | 0.07 ** | -0.09 ** | -0.02 | -0.15 ** | -0.09 ** | 0.33 ** | -0.20 ** | -0.20 ** | -0.10 ** | -0.14 ** | -0.21 ** | 0.24 | 0.09 | -0.23 ** | -0.11 ** | 0.26 | -0.17 ** | -0.02 | 0.02 | 0.14 ** |
|  | 0.60 ** | 0.47 ** | 0.36 ** | 1 | -0.03 | -0.15 ** | 0.18 ** | -0.02 | 0.08 ** | -0.11 ** | -0.09 ** | 0.33 ** | -0.21 ** | -0.19 ** | -0.08 ** | -0.14 ** | -0.20 ** | 0.18 ** | 0.09 ** | -0.22 ** | -0.14 ** | 0.39 ** | -0.20 ** | -0.12 ** | -0.10 ** | 0.23 ** |
|  | 0.02 | 0.13 ** | -0.11 ** | -0.03 | 1 | 0.49 ** | 0.53 ** | 0.37 ** | 0.45 ** | 0.46 ** | 0.34 ** | -0.04 ** | 0.25 ** | 0.24 ** | 0.22 ** | 0.01 | 0.17 ** | -0.18 ** | -0.20 ** | 0.39 ** | 0.13 ** | -0.23 ** | 0.22 ** | 0.11 ** | -0.23 ** | 0.23 ** |
|  | -0.04 * | -0.13 ** | -0.21 ** | -0.15 ** | 0.49 ** | 1 | 0.29 ** | 0.16 ** | 0.27 ** | 0.35 ** | 0.23 ** | -0.11 ** | 0.17 ** | 0.26 ** | 0.15 ** | 0.21 ** | 0.19 ** | -0.19 ** | -0.10 ** | 0.31 ** | 0.13 ** | -0.25 ** | 0.19 ** | 0.01 | -0.21 ** | 0.20 ** |
|  | 0.31 ** | 0.34 ** | 0.07 ** | 0.18 ** | 0.53 ** | 0.29 ** | 1 | 0.58 ** | 0.53 ** | 0.37 ** | 0.35 ** | 0.07 ** | 0.20 ** | 0.23 ** | 0.19 ** | -0.01 | 0.19 ** | -0.15 ** | -0.21 ** | 0.42 ** | 0.17 ** | -0.17 ** | 0.27 ** | 0.03 * | -0.18 ** | 0.42 ** |
|  | 0.09 ** | 0.16 ** | -0.09 ** | -0.02 | 0.37 ** | 0.16 ** | 0.58 ** | 1 | 0.61 ** | 0.43 ** | 0.50 ** | -0.13 ** | 0.34 ** | 0.31 ** | 0.23 ** | 0.01 | 0.32 ** | -0.22 ** | -0.23 ** | 0.53 ** | 0.16 ** | -0.21 ** | 0.32 ** | 0.08 ** | -0.07 ** | 0.16 ** |
|  | 0.13 ** | 0.24 ** | -0.02 | 0.08 ** | 0.45 ** | 0.27 ** | 0.53 ** | 0.61 ** | 1 | 0.46 ** | 0.42 ** | -0.05 ** | 0.35 ** | 0.29 ** | 0.26 ** | 0.02 | 0.28 ** | -0.14 ** | -0.13 ** | 0.49 ** | 0.12 ** | -0.12 ** | 0.27 ** | 0.09 ** | -0.08 ** | 0.17 ** |
|  | -0.06 ** | 0.01 | -0.15 ** | -0.11 ** | 0.46 ** | 0.35 ** | 0.37 ** | 0.43 ** | 0.46 ** | 1 | 0.56 ** | -0.24 ** | 0.55 ** | 0.44 ** | 0.31 ** | 0.09 ** | 0.39 ** | -0.31 ** | -0.23 ** | 0.50 ** | 0.16 ** | -0.26 ** | 0.26 ** | 0.08 ** | -0.08 ** | 0.17 ** |
|  | -0.02 | 0.01 | -0.09 ** | -0.09 ** | 0.34 ** | 0.23 ** | 0.35 ** | 0.50 ** | 0.42 ** | 0.56 ** | 1 | -0.18 ** | 0.36 ** | 0.32 ** | 0.22 ** | 0.04 * | 0.30 ** | -0.28 ** | -0.19 ** | 0.41 ** | 0.13 ** | -0.20 ** | 0.21 ** | 0.07 ** | -0.06 ** | 0.09 ** |
|  | 0.30 ** | 0.28 ** | 0.33 ** | 0.33 ** | -0.04 ** | -0.11 ** | 0.07 ** | -0.13 ** | -0.05 ** | -0.24 ** | -0.18 ** | 1 | -0.29 ** | -0.25 ** | -0.03 | -0.06 ** | -0.27 ** | 0.36 ** | 0.18 ** | -0.30 ** | -0.12 ** | 0.24 ** | -0.16 ** | -0.05 ** | -0.13 ** | 0.06 ** |
|  | -0.17 ** | -0.10 ** | -0.20 ** | -0.21 ** | 0.25 ** | 0.17 ** | 0.20 ** | 0.34 ** | 0.35 ** | 0.55 ** | 0.36 ** | -0.29 ** | 1 | 0.55 ** | 0.39 ** | 0.10 ** | 0.34 ** | -0.31 ** | -0.17 ** | 0.54 ** | 0.13 ** | -0.22 ** | 0.24 ** | 0.03 | 0.02 | 0.00 |
|  | -0.13 ** | -0.10 ** | -0.20 ** | -0.19 ** | 0.24 ** | 0.26 ** | 0.23 ** | 0.31 ** | 0.29 ** | 0.44 ** | 0.32 ** | -0.25 ** | 0.55 ** | 1 | 0.45 ** | 0.24 ** | 0.49 ** | -0.34 ** | -0.22 ** | 0.48 ** | 0.18 ** | -0.23 ** | 0.25 ** | -0.05 ** | 0.00 | 0.06 ** |
|  | -0.03 | 0.02 | -0.10 ** | -0.08 ** | 0.22 ** | 0.15 ** | 0.19 ** | 0.23 ** | 0.26 ** | 0.31 ** | 0.22 ** | -0.03 | 0.39 ** | 0.45 ** | 1 | 0.17 ** | 0.27 ** | -0.07 ** | -0.06 ** | 0.30 ** | 0.07 ** | -0.13 ** | 0.16 ** | 0.01 | -0.09 ** | -0.04 * |
|  | -0.06 ** | -0.14 ** | -0.14 ** | -0.14 ** | 0.01 | 0.21 ** | -0.01 | 0.01 | 0.02 | 0.09 ** | 0.04 * | -0.06 ** | 0.10 ** | 0.24 ** | 0.17 ** | 1 | 0.20 ** | -0.05 ** | 0.03 | 0.10 ** | 0.04 * | -0.09 ** | 0.08 ** | -0.14 ** | -0.03 | -0.10 ** |
|  | -0.12 ** | -0.14 ** | -0.21 ** | -0.20 ** | 0.17 ** | 0.19 ** | 0.19 ** | 0.32 ** | 0.28 ** | 0.33 ** | 0.30 ** | -0.27 ** | 0.34 ** | 0.49 ** | 0.27 ** | 0.20 ** | 1 | -0.38 ** | -0.19 ** | 0.49 ** | 0.15 ** | -0.2 4** | 0.28 ** | 0.04 * | 0.02 | 0.01 |
|  | 0.14 ** | 0.16 ** | 0.24 ** | 0.18 ** | -0.18 ** | -0.19 ** | -0.15 ** | -0.22 ** | -0.14 ** | -0.31 ** | -0.28 ** | 0.36 ** | -0.31 ** | -0.34 ** | -0.07 ** | -0.05 ** | -0.38 ** | 1 | 0.26 ** | -0.38 ** | -0.18 ** | 0.2 9** | -0.22 ** | -0.02 | -0.01 | -0.14 ** |
|  | 0.04 * | 0.00 | 0.09 ** | 0.09 ** | -0.20 ** | -0.10 ** | -0.21 ** | -0.23 ** | -0.13 ** | -0.23 ** | -0.19 ** | 0.18 ** | -0.17 ** | -0.22 ** | -0.06 ** | 0.03 | -0.19 ** | 0.26 ** | 1 | -0.32 ** | -0.22 ** | 0.27 ** | -0.31 ** | -0.02 | -0.04 * | -0.25 ** |
|  | -0.10 ** | -0.07 ** | -0.23 ** | -0.22 ** | 0.39 ** | 0.31 ** | 0.42 ** | 0.53 ** | 0.49 ** | 0.50 ** | 0.41 ** | -0.30 ** | 0.54 ** | 0.48 ** | 0.30 ** | 0.10 ** | 0.49 ** | -0.38 ** | -0.32 ** | 1 | 0.25 ** | -0.37 ** | 0.45 ** | 0.10 ** | -0.04 ** | 0.15 ** |
|  | -0.06 ** | -0.10 ** | -0.11 ** | -0.14 ** | 0.13 ** | 0.13 ** | 0.17 ** | 0.16 ** | 0.12 ** | 0.16 ** | 0.13 ** | -0.12 ** | 0.13 ** | 0.18 ** | 0.07 ** | 0.04 | 0.15 ** | -0.18 ** | -0.22 ** | 0.25 ** | 1 | -0.47 ** | 0.37 ** | -0.10 ** | -0.02 | 0.16 ** |
|  | 0.24 ** | 0.30 ** | 0.26 ** | 0.39 ** | -0.23 ** | -0.25 ** | -0.17 ** | -0.21 ** | -0.12 ** | -0.26 ** | -0.20 ** | 0.24 ** | -0.22 ** | -0.23 ** | -0.13 ** | -0.09 ** | -0.24 ** | 0.29 ** | 0.27 ** | -0.37 ** | -0.47 ** | 1 | -0.45 ** | -0.10 ** | 0.22 ** | -0.10 ** |
|  | -0.03 * | -0.05 ** | -0.17 ** | -0.20 ** | 0.22 ** | 0.19 ** | 0.27 ** | 0.32 ** | 0.27 ** | 0.26 ** | 0.21 ** | -0.16 ** | 0.24 ** | 0.25 ** | 0.16 ** | 0.08 ** | 0.28 ** | -0.22 ** | -0.31 ** | 0.45 ** | 0.37 ** | -0.45 ** | 1 | 0.04 ** | -0.03 | 0.20 ** |
|  | -0.14 ** | 0.02 | -0.02 | -0.12 ** | 0.11 ** | 0.01 | 0.03 * | 0.08 ** | 0.09 ** | 0.08 ** | 0.07 ** | -0.05 ** | 0.03 | -0.05 ** | 0.01 | -0.14 ** | 0.04 * | -0.02 | -0.02 | 0.10 ** | -0.10 ** | -0.10 ** | 0.04 ** | 1 | -0.05 ** | -0.08 ** |
|  | -0.11 ** | -0.02 | 0.02 | -0.10 ** | -0.23 ** | -0.21 ** | -0.18 ** | -0.07 ** | -0.08 ** | -0.09 ** | -0.06 ** | -0.13 ** | 0.02 | 0.00 | -0.09 ** | -0.03 * | 0.02 | -0.01 | -0.04 * | -0.04 ** | -0.02 | 0.22** | -0.03 | -0.05 ** | 1 | -0.14 ** |
|  | 0.30 ** | 0.28 ** | 0.14 ** | 0.23 ** | 0.23 ** | 0.20 ** | 0.42 ** | 0.16 ** | 0.17 ** | 0.17 ** | 0.09 ** | 0.06 ** | 0.00 | 0.06 ** | -0.04 * | -0.10 ** | 0.01 | -0.14 ** | -0.25 ** | 0.15 ** | 0.16 ** | -0.10 ** | 0.20 ** | -0.08 ** | -0.14 ** | 1 |

*P<0.05; **P<0.001

**Legend of Items:**

1. Quantitative Demands
2. Emotional Demands
3. Demands for hiding emotions
4. Work-Privacy Conflict
5. Influence at work
6. Degree of freedom at work
7. Possibilities for development
8. Meaning of work
9. Workplace commitment
10. Predictability
11. Role clarity
12. Role conflicts
13. Quality of leadership
14. Social Support
15. Feedback
16. Social relations
17. Sense of community
18. Mobbing (1-item)
19. Job insecurity

Outcomes:

1. Job satisfaction
2. General health (1-item)
3. Copenhagen Burnout Inventory
4. Satisfaction with Life Scale

Personal characteristics:

1. Age
2. Sex
3. Socioeconomic Status (SES)

**Table S4** Correlation matrix (Pearson correlation coefficients) for COPSOQ dimensions and outcomes (shaded in gray) between baseline and follow-up

| **Follow-up** | Quantitative Demands | Emotional Demands | Demands for hiding (emotions) | Work-privacy conflict | Influence at work | Degree of freedom at work | Possibilities for development | Meaning of work | Workplace commitment | Predictability | Role clarity | Role conflicts | Quality of Leadership | Social Support | Feedback | Social relations | Sense of community | Mobbing (1 Item) | Job insecurity Job | Job satisfaction | General health (1 Item) | Copenhagen Burnout Inventory | Satisfaction with Life Scale |
| --- | --- | --- | --- | --- | --- | --- | --- | --- | --- | --- | --- | --- | --- | --- | --- | --- | --- | --- | --- | --- | --- | --- | --- |
| **Baseline** | **1** | **2** | **3** | **4** | **5** | **6** | **7** | **8** | **9** | **10** | **11** | **12** | **13** | **14** | **15** | **16** | **17** | **18** | **19** | **20** | **21** | **22** | **23** |
| **1** | 0.63 ** | 0.33 ** | 0.24 ** | 0.43 ** | 0.07 ** | 0.00 ** | 0.25 ** | 0.04 | 0.11 ** | -0.02 | -0.02 ** | 0.26 ** | -0.11 ** | -0.11 ** | -0.03 | -0.07 ** | -0.08 ** | 0.10 ** | 0.06 ** | -0.07 ** | -0.08 ** | 0.15 ** | 0.00 |
| **2** | 0.32 ** | 0.62 ** | 0.34 ** | 0.35 | 0.17 ** | -0.09 ** | 0.29 ** | 0.11 ** | 0.20* * | 0.04 * | 0.01 | 0.24 ** | -0.06 * | -0.08 ** | 0.02 ** | -0.12 ** | -0.11 ** | 0.11 ** | 0.03 | -0.03 | -0.09 | 0.22 ** | -0.02 |
| **3** | 0.24 ** | 0.33 ** | 0.55 ** | 0.27 ** | -0.09 ** | -0.16 ** | 0.05 * | -0.05 * | -0.01 | -0.14 ** | -0.09 ** | 0.28 ** | -0.14 ** | -0.18 ** | -0.09 ** | -0.12 ** | -0.15 ** | 0.15 ** | 0.13 | -0.16 | -0.08 ** | 0.20 ** | -0.11 ** |
| **4** | 0.43 ** | 0.35 ** | 0.29 ** | 0.62 ** | 0.02 | -0.10 ** | 0.17 ** | -0.01 | 0.09 ** | -0.06 * | -0.06 | 0.26 ** | -0.15 ** | -0.16 ** | -0.07 ** | -0.11 ** | -0.14 ** | 0.13 ** | 0.11 ** | -0.15 ** | -0.11 ** | 0.24 ** | -0.14 ** |
| **5** | 0.01 | 0.10 ** | -0.09 ** | 0.02 | 0.70 ** | 0.39 ** | 0.45 ** | 0.29 ** | 0.39 ** | 0.41 ** | 0.29 ** | 0.00 | 0.18 ** | 0.17 ** | 0.18 ** | 0.01 | 0.12 ** | -0.11 ** | -0.14 ** | 0.29 ** | 0.10 ** | -0.16 ** | 0.16 ** |
| **6** | 0.00 | -0.10 ** | -0.18 ** | -0.0 8** | 0.42 ** | 0.74 ** | 0.27 ** | 0.12 ** | 0.19 ** | 0.30 ** | 0.18 ** | -0.05 ** | 0.12 ** | 0.19 ** | 0.09 ** | 0.18 ** | 0.11 ** | -0.12 ** | -0.07 ** | 0.22 ** | 0.11 ** | -0.18 ** | 0.13 ** |
| **7** | 0.23 ** | 0.30 ** | 0.06 ** | 0.16 ** | 0.47 ** | 0.26 ** | 0.71 ** | 0.40 ** | 0.41 ** | 0.32 ** | 0.30 ** | 0.07 ** | 0.15 ** | 0.15 ** | 0.13 ** | -0.03 | 0.15 ** | -0.08 ** | -0.16 ** | 0.30 ** | 0.10 ** | -0.15 ** | 0.24 ** |
| **8** | 0.04 | 0.14 ** | -0.05 * | -0.01 | 0.31 ** | 0.12 ** | 0.43 ** | 0.58 ** | 0.41 ** | 0.32 ** | 0.38 ** | -0.11 ** | 0.24 ** | 0.22 ** | 0.15 ** | 0.01 | 0.25 ** | -0.14 ** | -0.23 ** | 0.37 ** | 0.13 ** | -0.18 ** | 0.30 ** |
| **9** | 0.09 ** | 0.21 ** | 0.01 | 0.10 ** | 0.42 ** | 0.23 ** | 0.42 ** | 0.42 ** | 0.66 ** | 0.38 ** | 0.31 ** | -0.03 | 0.23 ** | 0.21 ** | 0.20 ** | 0.01 | 0.21 ** | -0.10 ** | -0.12 ** | 0.33 ** | 0.08 ** | -0.09 ** | 0.24 ** |
| **10** | -0.06 * | 0.03 | -0.10 ** | -0.05 * | 0.41 ** | 0.29 ** | 0.28 ** | 0.28 ** | 0.36 ** | 0.56 ** | 0.37 ** | -0.20 ** | 0.31 ** | 0.28 ** | 0.20 ** | 0.06 ** | 0.22 ** | -0.19 ** | -0.19 ** | 0.35 ** | 0.14 ** | -0.21 ** | 0.20 ** |
| **11** | -0.03 | 0.01 | -0.07 ** | -0.04 * | 0.28 ** | 0.17 ** | 0.27 ** | 0.35 ** | 0.31 ** | 0.39 ** | 0.56 ** | -0.18 ** | 0.20 ** | 0.22 ** | 0.13 ** | 0.05 * | 0.23 ** | -0.17 ** | -0.16 ** | 0.31 ** | 0.10 ** | -0.17 ** | 0.20 ** |
| **12** | 0.23 ** | 0.23 ** | 0.26 ** | 0.25 ** | -0.05 * | -0.07 * | 0.06 ** | -0.11 ** | -0.05 * | -0.20 ** | -0.16 ** | 0.49 ** | -0.18 ** | -0.18 ** | -0.02 | -0.04 * | -0.19 ** | 0.24 ** | 0.16 ** | -0.21 ** | -0.12 ** | 0.17 ** | -0.11 ** |
| **13** | -0.13 ** | -0.09 ** | -0.12 ** | -0.11 ** | 0.17 ** | 0.12 ** | 0.14 ** | 0.22 ** | 0.24 ** | 0.33 ** | 0.22 ** | -0.18 ** | 0.44 ** | 0.32 ** | 0.28 ** | 0.07 ** | 0.23 ** | -0.18 ** | -0.15 ** | 0.33 ** | 0.12 ** | -0.17 ** | 0.17 ** |
| **14** | -0.07 ** | -0.04 | -0.12 ** | -0.12 ** | 0.20 ** | 0.19 ** | 0.20 ** | 0.22 ** | 0.22 ** | 0.30 ** | 0.24 ** | -0.15 ** | 0.29 ** | 0.50 ** | 0.26 ** | 0.18 ** | 0.31 ** | -0.20 ** | -0.18 ** | 0.29 ** | 0.14 ** | -0.16 ** | 0.19 ** |
| **15** | -0.04 * | 0.00 | -0.07 ** | -0.06 * | 0.20 ** | 0.11 ** | 0.14 ** | 0.16 ** | 0.20 ** | 0.19 ** | 0.17 ** | -0.02 | 0.21 ** | 0.25 ** | 0.45 ** | 0.11 ** | 0.18 ** | -0.03 | -0.06 * | 0.18 ** | 0.04 * | -0.10 ** | 0.11 ** |
| **16** | -0.04 | -0.10 ** | -0.12 ** | -0.11 ** | 0.01 | 0.17 ** | -0.03 | -0.00 | -0.02 | 0.04 | 0.05 * | -0.05 * | 0.11 ** | 0.20 ** | 0.12 ** | 0.54 ** | 0.14 ** | -0.03 | 0.01 | 0.04 * | 0.05 * | -0.05 * | 0.01 |
| **17** | -0.10 ** | -0.08 ** | -0.14 ** | -0.15 ** | 0.15 ** | 0.14 ** | 0.15 ** | 0.25 ** | 0.21 ** | 0.24 ** | 0.24 ** | -0.19 ** | 0.23 ** | 0.34 ** | 0.20 ** | 0.16 ** | 0.47 ** | -0.23 ** | -0.16 ** | 0.32 ** | 0.13 ** | -0.18 ** | 0.25 ** |
| **18** | 0.12 ** | 0.14 ** | 0.14 ** | 0.14 ** | -0.13 ** | -0.12 ** | -0.11 ** | -0.15 ** | -0.08 ** | -0.20 ** | -0.18 ** | 0.23 ** | -0.17 ** | -0.21 ** | -0.07 * | -0.00 | -0.26 ** | 0.37 ** | 0.22 ** | -0.24 ** | -0.16 ** | 0.20 ** | -0.18 ** |
| **19** | 0.04 * | -0.00 | 0.07 * | 0.06 * | -0.20 ** | -0.10 ** | -0.25 ** | -0.24 ** | -0.16 ** | -0.23 ** | -0.20 ** | 0.15 ** | -0.16 ** | -0.19 ** | -0.06 * | 0.06 * | -0.17 ** | 0.21 ** | 0.58 ** | -0.27 ** | -0.18 ** | 0.23 ** | -0.27 ** |
| **20** | -0.07 ** | -0.03 | -0.14 ** | -0.15 ** | 0.32 ** | 0.24 ** | 0.33 ** | 0.35* * | 0.33 ** | 0.36 ** | 0.31 ** | -0.19 ** | 0.33 ** | 0.32 ** | 0.21 ** | 0.07 * | 0.33 ** | -0.23 ** | -0.24 ** | 0.49 ** | 0.19 ** | -0.26 ** | 0.35 ** |
| **21** | -0.03 | -0.07 ** | -0.08 ** | -0.13 ** | 0.09 ** | 0.10 ** | 0.12 ** | 0.13 ** | 0.10 ** | 0.15 ** | 0.13 ** | -0.10 ** | 0.12 ** | 0.12 ** | 0.05 * | 0.03 | 0.14 ** | -0.14 ** | -0.16 ** | 0.19 ** | 0.49 ** | -0.33 ** | 0.29 ** |
| **22** | 0.22 ** | 0.25 ** | 0.22 ** | 0.31 ** | -0.17 ** | -0.20 ** | -0.12 ** | -0.18 ** | -0.09 ** | -0.20 ** | -0.19 ** | 0.19 ** | -0.15 ** | -0.17 ** | -0.09 ** | -0.06 * | -0.22 ** | 0.21 ** | 0.22 ** | -0.28 ** | -0.34 ** | 0.68 ** | -0.37 ** |
| **23** | -0.04 * | -0.02 | -0.14 ** | -0.17 ** | 0.18 ** | 0.16 ** | 0.22 ** | 0.26 ** | 0.20 ** | 0.23* * | 0.19 ** | -0.13 ** | 0.22 ** | 0.21 ** | 0.13 ** | 0.03 | 0.23 ** | -0.19 ** | -0.26 ** | 0.35 ** | 0.26 ** | -0.35 ** | 0.68 ** |

*P<0.05; **P<0.001

1. Quantitative Demands
2. Emotional Demands
3. Demands for hiding emotions
4. Work-Privacy Conflict
5. Influence at work
6. Degree of freedom at work
7. Possibilities for development
8. Meaning of work
9. Workplace commitment
10. Predictability
11. Role clarity
12. Role conflicts
13. Quality of leadership
14. Social Support
15. Feedback
16. Social relations
17. Sense of community
18. Mobbing (1 Item)
19. Job insecurity

Outcomes:

1. Job satisfaction
2. General health (1 Item)
3. Copenhagen Burnout Inventory
4. Satisfaction with Life Scale

**Table S5** Correlation matrix (Pearson correlation coefficients) for ERI dimensions, outcomes (shaded in gray), age and sex at baseline

|  |  | Effort (6 Items) | Reward (11 Items) | Effort/Reward Quotient | Overcommitment  (6 Items) | Effort (0-100) | Reward (0-100) | Effort/Reward (0-100) | Overcommitment (0-100) | Job Satisfaction | General health  (1 Item) | Copenhagen Burnout Inventory (CBI) | Satisfaction with life scale (SWLS) | Age | Sex | SES |
| --- | --- | --- | --- | --- | --- | --- | --- | --- | --- | --- | --- | --- | --- | --- | --- | --- |
|  |  | **1** | **2** | **3** | **4** | **5** | **6** | **7** | **8** | **9** | **10** | **11** | **12** | **13** | **14** | **15** |
| Effort (6 Items) | **1** | 1.00 | -0.29 ** | 0.86 | 0.55 | 1.00 | -0.29 | 0.77 ** | 0.55 ** | -0.24 ** | -0.17 | 0.36 ** | -0.16 ** | -0.09 ** | -0.11 ** | 0.10 ** |
| Reward (11 Items) | **2** | -0.29 ** | 1.00 | -0.67 ** | -0.30 ** | -0.29 ** | 1.00 | -0.59 ** | -0.30 ** | 0.66 ** | 0.27 ** | -0.38 ** | 0.41 ** | 0.07 ** | -0.02 | 0.07 ** |
| ERI-Quotient (Effort/Reward) | **3** | 0.86 ** | -0.67 ** | 1.00 | 0.54 ** | 0.86 ** | -0.67 ** | 0.94 ** | 0.54 ** | -0.47 ** | -0.26 ** | 0.43 ** | -0.30 ** | -.06 ** | -0.05 ** | 0.03 |
| Overcommitment  (6 Items) | **4** | 0.55 ** | -0.30 ** | 0.54 ** | 1.00 | 0.55 ** | -0.29 ** | 0.46 ** | 1.00 | -0.26 ** | -0.23 ** | 0.44 ** | -0.24 ** | 0.00 | -0.03 * | 0.11 ** |
| Effort (0-100) | **5** | 1.00 | -0.29 ** | 0.86 ** | 0.55 ** | 1.00 | -0.29 ** | 0.77 ** | 0.55 ** | -0.25 ** | -0.17 ** | 0.36 ** | -0.16 ** | -0.09 ** | -0.11 ** | 0.9 ** |
| Reward (0-100) | **6** | -0.29 ** | 1.00 | -0.67 ** | -0.29 ** | -0.29 ** | 1.00 | -0.60 ** | -0.30 ** | 0.65 | 0.27 ** | -0.38 ** | 0.41 ** | 0.09 ** | -0.01 | 0.7 ** |
| ERI Quotient  (Effort/Reward, 100 Scale) | **7** | 0.77 ** | -0.59 ** | 0.94 ** | 0.46 ** | 0.77 ** | -0.60 ** | 1.00 | 0.46 ** | -0.40 ** | -0.22 ** | 0.36 ** | -0.26 ** | -0.07 ** | -0.05 ** | 0.02 |
| Overcommitment (0-100) | **8** | 0.55 ** | -0.30 ** | 0.54 ** | 1.00 | 0.55 ** | -0.30 ** | 0.46 ** | 1.00 | -0.27 ** | -0.23 ** | 0.44 ** | -0.25 ** | 0.00 | -0.04 * | 0.11 ** |
| Job Satisfaction | **9** | -0.24 ** | 0.66 ** | -0.47 ** | -0.26 ** | -0.25 ** | 0.65 ** | -0.40 ** | -0.27 ** | 1.00 | 0.28 ** | -0.39 ** | 0.46 ** | 0.09 ** | -0.02 | 0.13 ** |
| General Health (1 Item) | **10** | -0.17 ** | 0.27 ** | -0.26 ** | -0.23 ** | -0.17 ** | 0.27 ** | -0.22 ** | -0.23 ** | 0.28 ** | 1.00 | -0.48 ** | 0.38 ** | -.11 ** | 0.01 | 0.16 ** |
| Copenhagen Burnout Inventory (CBI) | **11** | 0.36 ** | -0.38 ** | 0.43 ** | 0.44 ** | 0.36 ** | -0.38 ** | 0.36 ** | 0.44 ** | -0.39 ** | -0.48 ** | 1.00 | -0.48 ** | -.09 ** | .20 ** | -0.12 ** |
| Satisfaction with life scale (SWLS) | **12** | -0.16 ** | 0.41 ** | -0.30 ** | -0.24 ** | -0.16 ** | 0.41 ** | -0.26 ** | -0.25 ** | 0.46 ** | 0.38 ** | -0.48 ** | 1.00 | 0.04 ** | -.01 | 0.17 ** |
| Age | **13** | -0.09 ** | 0.07 ** | -0.06 ** | 0.00 | -0.09 ** | 0.09 ** | -0.07 ** | 0.00 | 0.09 ** | -0.11 ** | -0.09 ** | 0.04 ** | 1 | -.07 ** | -0.05 ** |
| Sex | **14** | -0.11 ** | -0.02 | -0.05 ** | -0.03 * | -0.11 ** | -0.01 | -0.05 ** | -0.04 ** | -0.02 | 0.01 | 0.20 ** | -0.01 | -0.07 ** | 1 | -0.14 ** |
| SES | **15** | 0.10 ** | 0.07 ** | 0.03 | 0.11 ** | 0.09 ** | 0.07 ** | 0.02 | 0.11 ** | 0.13 ** | 0.16 ** | -0.12 ** | 0.17 ** | -0.05 ** | -0.14 ** | 1.00 |

*P<0.05; **P<0.001

**Table S6** Correlation matrix (Pearson correlation coefficients) for between ERI dimensions at baseline and follow-up.

| **Baseline** | **Follow-Up** | | | | | | | | | | | |
| --- | --- | --- | --- | --- | --- | --- | --- | --- | --- | --- | --- | --- |
|  | Effort (6 Items) | Reward  (11 Items) | ERI-Quotient (Effort/Reward) | Overcommitment  (6 Items) | Effort  (0-100) | Reward  (0-100) | ERI-Quotient (Effort/Reward, 100 Scale) | Overcommitment  (0-100) | Job Satisfaction | General health  (1 Item) | Copenhagen Burnout Inventory (CBI) | Satisfaction with life scale (SWLS) |
| Effort (6 Items) | 0.58** | -0.20** | 0.50** | 0.42** | 0.58** | -0.18** | 0.43** | 0.42** | -0.15** | -0.13** | 0.24** | -0.11** |
| Reward (11 Items) | -0.21** | 0.57** | -0.40** | -0.21** | -0.21** | 0.56** | -0.32** | -0.21** | 0.42** | 0.22** | -0.31** | 0.35** |
| ERI-Quotient (Effort/Reward) | 0.52** | -0.42** | 0.58** | 0.39** | 0.52** | -0.41** | 0.48** | 0.39** | -0.31** | -0.21** | 0.31** | -0.25** |
| Overcommitment  (6 Items) | 0.39** | -0.21** | 0.37** | 0.65** | 0.39** | -0.19** | 0.32** | 0.65** | -0.17** | -0.18** | 0.31** | -0.20** |
| Effort (0-100) | 0.58** | -0.20** | 0.50** | 0.41** | 0.58** | -0.19** | 0.43** | 0.42** | -0.15** | -0.14** | 0.23** | -0.11** |
| Reward (0-100) | -0.21** | 0.56** | -0.39** | -0.20** | -0.20** | 0.56** | -0.32** | -0.20** | 0.43** | 0.22** | -0.30** | 0.35** |
| ERI-Quotient (Effort/Reward, 100 Scale) | 0.49** | -0.37** | 0.54** | 0.36** | 0.48** | -0.36** | 0.47** | 0.36** | -0.28** | -0.19** | 0.28** | -0.21** |
| Overcommitment (0-100) | 0.39** | -0.21** | 0.36** | 0.65** | 0.39** | -0.20** | 0.31** | 0.65** | -0.17** | -0.18** | 0.31** | -0.19** |
| Job satisfaction | -0.18** | 0.42** | -0.30** | -0.17** | -0.18** | 0.43** | -0.26** | -0.17** | 0.54** | 0.23** | -0.29** | 0.38** |
| General health  (1 Item) | -0.12** | 0.19** | -0.18** | -0.17** | -0.12** | 0.18** | -0.15** | -0.17** | 0.20** | 0.53** | -0.37** | 0.32** |
| Copenhagen Burnout Inventory (CBI) | 0.26** | -0.30** | 0.31** | 0.34** | 0.26** | -0.30** | 0.27** | 0.34** | -0.29** | -0.37** | 0.67** | -0.40** |
| Satisfaction with life scale (SWLS) | -0.11** | 0.33** | -0.21** | -0.18** | -0.11** | 0.33** | -0.18** | -0.18** | 0.37** | 0.31** | -0.36** | 0.70** |

*P<0.05; **P<0.001

**Table S7** ERI cross-sectional multivariable linear regression models (factors shown in the order of selection), and adjusted for age, sex, and socioeconomic status. Due to the model selection procedure, the models selected at t0 sometimes differ from the models selected at t1.

|  | **ERI (t0)** |  |  | **ERI (t1)** |  |  |
| --- | --- | --- | --- | --- | --- | --- |
| **Outcomes at t0 or t1** | **Scales** | **Beta (SE)** | **R^2^** | **Scales** | **Beta (SE)** | **R^2^** |
| **Job satisfaction** | Reward | 0.674 (0.016) | 0.44 | Reward | 0.661 (0.016) | 0.44 |
| **at t0 or t1** | Effort | -0.074 (0.018) |  | Overcommitment | -0.073 (0.011) |  |
|  | Overcommitment | -0.057 (0.010) |  |  |  |  |
|  | Ratio | 0.036 (0.010) |  |  |  |  |
|  | N=4208; 10 ^a^ |  |  | N=3058; 12 ^a^ |  |  |
| **General health** | Reward | 0.242 (0.017) | 0.14 | Reward | 0.228 (0.020) | 0.12 |
| **at t0 or t1** | Overcommitment | -0.141 (0.012) |  | Overcommitment | -0.138 (0.013) |  |
|  | N=4219; 43 ^a^ |  |  | N=3062; 29 ^a^ |  |  |
| **Copenhagen Burnout Inventory Inventory** | Reward | -0.304 (0.020) | 0.34 | Reward | -0.335 (0.020) | 0.35 |
|  | Overcommitment  E | 0.253 (0.013) |  | Overcommitment  E | 0.272 (0.016) |  |
| **at t0 or t1** | Effort | 0.191 (0.022) |  | Effort | 0.125 (0.019) |  |
|  | Ratio  N | -0.037 (0.013) |  | N |  |  |
|  | N=4191; 11 ^a^ |  |  | N=3036; 18 ^a^ |  |  |
| **Satisfaction with Life Scale** | Reward | 0.472 (0.021) | 0.22 | Reward | 0.522 (0.025) | 0.22 |
| **at t0 or t1** | Overcommitment | -0.153 (0.013) |  | Overcommitment | -0.130 (0.015) |  |
|  | Ratio | 0.026 (0.010) |  | Ratio | 0.035 (0.011) |  |
|  | N=4182; 26 ^a^ |  |  | N=3029; 23 ^a^ |  |  |

*^a^ number of cases with residuals 3 or more ±SD from the prediction
Results of the forward stepwise regression analyses. Four health and work related outcomes at the baseline (t_0_) or at the 5-year follow–up (t_1_) were predicted by the ERI at the same survey time); R^2^ = Proportion of the variance explained by the model; SE Standard Error; N valid case number (fluctuates due to missing values)*

**Table S8** ERI prospective linear multivariable regression models (factors shown in the order of selection), and adjusted for age, sex, and socioeconomic status.

|  | **ERI (t0)** | | | **ERI (t0)** adjusted for t0 values of outcomes | | |
| --- | --- | --- | --- | --- | --- | --- |
| **Outcomes at t1** | **Scales** | **Beta (SE)** | **R^2^** | **Scales** | **Beta (SE)** | **R^2^** |
| **Job satisfaction at t1** | Reward | 0.430 (0.018) | 0.21 | Reward | 0.142 (0.022) | 0.32 |
|  | Overcommitment | -0.066 (0.013) |  | Overcommitment | -0.039 (0.012) |  |
|  |  |  |  |  |  |  |
|  | N=3039; 17 ^a^ | |  | N=3027; 21 ^a^ |  |  |
| **General health at t1** | Reward | 0.194 (0.020) | 0.09 | Reward | 0.085 (0.018) | 0.29 |
|  | Overcommitment | -0.112 (0.014) |  | Overcommitment | -0.048 (0.012) |  |
|  |  |  |  |  |  |  |
|  | N=3050; 30 ^a^ |  |  | N=3049; 52 ^a^ |  |  |
| **Copenhagen Burnout** | Reward | -0.260 (0.022) | 0.22 | Reward | -0. 099 (0.018) | 0.46 |
| **Inventory at t1** | Overcommitment  E | 0.204 (0.017) |  |  |  |  |
|  | Effort | 0.099 (0.021) |  |  |  |  |
|  | N |  |  |  |  |  |
|  | N=3027; 11 ^a^ |  |  | N=2996; 22 ^a^ |  |  |
| **Satisfaction with Life Scale** | Reward | 0.425 (0.026) | 0.16 | Reward | 0.107 (0.018) | 0.50 |
| **at t1** | Overcommitment | -0.130 (0.016) |  |  |  |  |
|  | Ratio | 0.035 (0.013) |  |  |  |  |
|  |  |  |  |  |  |  |
|  | N=3016; 24 ^a^ |  |  | N=2993; 35 ^a^ |  |  |

*^a^ number of cases with residuals 3 or more ±SD from the prediction
Results of the forward stepwise regression analyses. Four health and work related outcomes at the 5-year follow–up (t_1_) were predicted prospectively by the ERI values at baseline (t_0_); R^2^ = Proportion of the variance explained by the model; SE Standard Error; N valid case number (fluctuates due to missing values)*

**Table S9** COPSOQ cross-sectional multivariable linear regression models (factors shown in the order of selection), and adjusted for age, sex, and socioeconomic status.

|  | **COPSOQ (t0)** | | | **COPSOQ (t1)** | | |
| --- | --- | --- | --- | --- | --- | --- |
| **Outcomes at t0 or t1** | **Scales** | **Beta (SE)** | **R^2^** | **Scales** | **Beta (SE)** | **R^2^** |
| **Job satisfaction** |  |  | 0.52 |  |  | 0.57 |
| **at t0 or t1** | Quality of leadership | 0.202 (0.009) |  | Quality of leadership | 0.180 (0.011) |  |
|  | Meaning of work | 0.235 (0.011) |  | Meaning of work | 0.225 (0.013) |  |
|  | Sense of community | 0.205 (0.012) |  | Sense of community | 0.196 (0.014) |  |
|  | Degree of freedom at work | 0.077 (0.008) |  | Predictability | 0.120 (0.013) |  |
|  | Job Insecurity | -0.089 (0.009) |  | Work-Privacy conflict | -0.081 (0.008) |  |
|  | N=3163; 11 ^a^ |  |  | N=2308; 10 ^a^ |  |  |
| **General health** |  |  | 0.13 |  |  | 0.12 |
| **at t0 or t1** | Job insecurity | -0.103 (0.015) |  | Predictability | 0.050 (0.018) |  |
|  | Possibilities for development | 0.134 (0.017) |  | Work-Privacy conflict | -0.119 (0.013) |  |
|  | Emotional demands | -0.075 (0.015) |  | Possibilities for development | 0.062 (0.019) |  |
|  | Work-Privacy conflict | -0.086 (0.012) |  | Sense of community | 0.098 (0.021) |  |
|  | Mobbing (1 Item) | -0.062 (0.014) |  | Job insecurity | -0.046 (0.016) |  |
|  | N=3150; 36 ^a^ |  |  | N=2298; 28 ^a^ |  |  |
| **Copenhagen Burnout** |  |  | 0.36 |  |  | 0.35 |
| **Inventory** | Work-Privacy conflict | 0.213 (0.011) |  | Work-Privacy conflict | 0.221 (0.014) |  |
| **at t0 or t1** | Possibilities for development | -0.156 (0.015) |  | Job insecurity | 0.153 (0.016) |  |
|  | Emotional demands | 0.177 (0.014) |  | Possibilities for development | -0.120 (0.020) |  |
|  | Job insecurity | 0.129 (0.013) |  | Emotional demands | 0.157 (0.017) |  |
|  | Mobbing (1 Item) | 0.100 (0.013) |  | Sense of community | -0.138 (0.021) |  |
|  | N=3163; 8 ^a^ |  |  | N=2310; 9 ^a^ |  |  |
| **Satisfaction with Life** |  |  | 0.23 |  |  | 0.22 |
| **Scale** | Job insecurity | -0.150 (0.015) |  | Meaning of work | 0.163 (0.021) |  |
| **at t0 or t1** | Meaning of work | 0.113 (0.020) |  | Job insecurity | -0.117 (0.017) |  |
|  | Work-Privacy conflict | -0.135 (0.011) |  | Work-Privacy Conflict | -0.127 (0.013) |  |
|  | Possibilities for development | 0.118 (0.019) |  | Sense of community | 0.129 (0.021) |  |
|  | Sense of community | 0.145 (0.018) |  | Possibilities for development | 0.065 (0.022) |  |
|  | N=3158; 32 ^a^ |  |  | N=2307; 27 ^a^ |  |  |

*^a^ number of cases with residuals 3 or more ±SD from the prediction*Results of the forward stepwise regression analyses. Four health and work related outcomes at the baseline (t_0_) or at the 5-year follow–up (t_1_) were predicted by the COPSOQ at the same survey time). R^2^ = Proportion of the variance explained by the model. SE Standard Error*; N valid case number (fluctuates due to missing values)*

**Table S10** COPSOQ prospective linear multivariable regression models (factors shown in the order of selection), and adjusted for age, sex, and socioeconomic status.

|  | **COPSOQ (t0)** | | | **COPSOQ (t0)** adjusted for t0 values of outcomes | | |
| --- | --- | --- | --- | --- | --- | --- |
| **Outcomes at t1** | **Scales** | **Beta (SE)** | **R^2^** | **Scales** | **Beta (SE)** | **R^2^** |
| **Job satisfaction at t1** |  |  | 0.24 |  |  | 0.28 |
|  | Meaning of work | 0.140 (0.017) |  | Job insecurity | -0.084 (0.014) |  |
|  | Sense of community | 0.160 (0.019) |  | Meaning of work | 0.078 (0.018) |  |
|  | Predictability | 0.089 (0.018) |  | Sense of community | 0.096 (0.019) |  |
|  | Job insecurity | -0.102 (0.015) |  | Predictability | 0.066 (0.016) |  |
|  | Quality of leadership | 0.085 (0.015) |  |  |  |  |
|  | N=2280; 16 ^a^ |  |  | N=2273; 22 ^a^ |  |  |
| **General health at t1** |  |  | 0.08 |  |  | 0.26 |
|  | Job insecurity | -0.081 (0.017) |  | Mobbing (1 Item) | -0.039 (0.015) |  |
|  | Mobbing (1 Item) | -0.061 (0.017) |  | Job insecurity | -0.035 (0.016) |  |
|  | Work-Privacy conflict | -0.068 (0.014) |  | Role conflict | -0.050 (0.017) |  |
|  | Possibilities for development | 0.064 (0.020) |  |  |  |  |
|  | Emotional demands | -0.052 (0.017) |  |  |  |  |
|  | N=2270; 23 ^a^ |  |  | N=2251; 33 ^a^ |  |  |
| **Copenhagen Burnout** |  |  | 0.22 |  |  | 0.47 |
| **Inventory at t1** | Work-Privacy conflict | 0.141 (0.015) |  | Job insecurity | 0.055 (0.015) |  |
|  | Job insecurity | 0.148 (0.018) |  | Demands for hiding emotions | 0.035 (0.012) |  |
|  | Possibilities for development | -0.104 (0.022) |  | Possibilities for development | -0.008 (0.017) |  |
|  | Emotional demands | 0.161 (0.019) |  |  |  |  |
|  | Predictability | -0.081 (0.019) |  |  |  |  |
|  | N=2295; 6 ^a^ |  |  | N=2291; 11 ^a^ |  |  |
| **Satisfaction with Life** |  |  | 0.17 |  |  | 0.47 |
| **Scale at t1** | Meaning of work | 0.130 (0.023) |  | Meaning of work | 0.052 (0.016) |  |
|  | Job insecurity | -0.132 (0.018) |  | Job insecurity | -0.043 (0.014) |  |
|  | Sense of community | 0.140 (0.22) |  | Sense of community | 0.061 (0.018) |  |
|  | Work-Privacy conflict | -0.089 (0.013) |  | Social relations | -0.027 (0.010) |  |
|  | Possibilities for development | 0.067 (0.023) |  |  |  |  |
|  | N=2294; 30 ^a^ |  |  | N=2286; 37 ^a^ |  |  |

*^a^ number of cases with residuals 3 or more ±SD from the prediction
Results of the forward stepwise regression analyses. Four health and work related outcomes at the 5-year follow–up (t_1_) were predicted by the COPSOQ prospectively (COPSOQ t_0_ 🡪 outcomes t_1_); R^2^ = Proportion of the variance explained by the model; SE Standard Error; N valid case number (fluctuates due to missing values)*
